# Endocrine islet β-cell subtypes with differential function are derived from biochemically distinct embryonic endocrine islet progenitors that are regulated by maternal nutrients

**DOI:** 10.21203/rs.3.rs-3946483/v1

**Published:** 2024-03-07

**Authors:** guoqiang Gu, Monica Brown, Verda Agan, Simone Nevills, Ruiying Hu, Alan Simmons, Yanwen Xu, Yilin Yang, Mahircan Yagan, Sadia Najam, Prasanna Dadi, Leesa Sampson, Mark Magnuson, David Jacobson, Ken Lau, Emily Hodges

**Affiliations:** Vanderbilt University; Vanderbilt University; Vanderbilt University; Vanderbilt University; Vanderbilt University Medical Center; Vanderbilt University; Vanderbilt University; Vanderbilty University School of Medicine; Vanderbilt University; Vanderbilt University; Vanderbilt University; Vanderbilt University; Vanderbilt University; Vanderbilt University; Vanderbilt University; Vanderbilt University

## Abstract

Endocrine islet b cells comprise heterogenous cell subsets. Yet when/how these subsets are produced and how stable they are remain unknown. Addressing these questions is important for preventing/curing diabetes, because lower numbers of b cells with better secretory function is a high risk of this disease. Using combinatorial cell lineage tracing, scRNA-seq, and DNA methylation analysis, we show here that embryonic islet progenitors with distinct gene expression and DNA methylation produce b-cell subtypes of different function and viability in adult mice. The subtype with better function is enriched for genes involved in vesicular production/trafficking, stress response, and Ca^2+^-secretion coupling, which further correspond to differential DNA methylation in putative enhancers of these genes. Maternal overnutrition, a major diabetes risk factor, reduces the proportion of endocrine progenitors of the b-cell subtype with better-function via deregulating DNA methyl transferase 3a. Intriguingly, the gene signature that defines mouse b-cell subtypes can reliably divide human cells into two sub-populations while the proportion of b cells with better-function is reduced in diabetic donors. The implication of these results is that modulating DNA methylation in islet progenitors using maternal food supplements can be explored to improve b-cell function in the prevention and therapy of diabetes.

## Introduction

Endocrine islet b cells regulate whole-body glucose homeostasis via glucose-stimulated-insulin secretion (GSIS). Insufficient GSIS results in diabetes, a metabolic disease that afflicts over 6% of the world population. Thus, understanding how each individual makes a sufficient number of b cells with specific levels of secretory function, known as functional b-cell mass, holds the key to prevent and to cure this disease.

The majority of b cells in adults are made through two pathways. During embryogenesis, islet progenitors that transiently express transcription factor (TF) Neurogenin3 (Neurog3 or Ngn3) differentiate into immature b cells (Gu et al., 2002). At late gestation and early postnatal stages, these cells proliferate and mature to make up the functional b-cell mass needed for physiology. Intriguingly, previous work has clearly established that adult b cells comprise cell subsets with differential levels of GSIS (Bader et al., 2016; Dorrell et al., 2016; Salomon and Meda, 1986), proliferation (Bader et al., 2016), metabolism (Pipeleers et al., 2017), and Ca^2+^ influx (Johnston et al., 2016). Yet when/how each b-cell subset is determined, how stable they are, and how altering the proportions of different b-cell subsets affect the risk of diabetes have not been directly tested.

A majority of published data are consistent with an idea that most reported b-cell subsets are not stable. They are observed largely due to two main reasons. First, b-cell differentiation, maturation, proliferation, senescence, and death are asynchronous, resulting in cells of different properties at time of studies (Aguayo-Mazzucato et al., 2017; Bader et al., 2016; Chiou et al., 2021; Finegood et al., 1995; Katsumoto et al., 2022; Piper et al., 2004; Ramond et al., 2017; Thompson et al., 2019; Zeng et al., 2017). For example, Bader et al. detected two b-cell subsets that do or do not express Wnt/planar cell polarity effector Fltp (or Cfap126) (Fltp^+^ and Fltp^−^) (Bader et al., 2016). The Fltp^−^ cells are less functional but over time they convert to more functional Fltp^+^cells, supporting them representing transient cell states (Bonner-Weir and Aguayo-Mazzucato, 2016). Second, GSIS invokes glucose metabolism, insulin biosynthesis, and stress responses. These variables, due to their stochastic nature, can create distinct b-cell subsets (Dominguez-Gutierrez et al., 2019; Fernandez-de-Cossio-Diaz et al., 2019; Xin et al., 2018).

In contrast, some reported b cell subsets are relatively stable. For example, a few “virgin b cells” that arise via postnatal a-ell transdifferentiation can retain their immaturity for months (Lee et al., 2021). Additionally, two recent studies suggest that differential DNA methylation in several enhancers and/or global histone methylation (H3K27me3) can mark relatively stable b-cell subtypes, whose proportions can be modified by the dosages of genes that render these modifications (Dror et al., 2023; Parveen et al., 2023). However, virgin b cells represent only a few percentage of all b cells (van der Meulen et al., 2017). Metabolic signaling have been shown to alter the DNA and histone methylation states in b cells (Avrahami and Kaestner, 2019; Avrahami et al., 2015; Brasacchio et al., 2009; Hall et al., 2018; Hall et al., 2019; Wortham et al., 2023). Thus, questions regarding the long-term stability of large b-cell subsets, in addition to stage when these heterogeneities are induced, remain to be answered.

Besides the issues on b-cell subset stability and origin, the causal relations between b-cell heterogeneity and diabetes are not known. Several studies have shown that the proportions of b-cell subsets with better-functionality are usually reduced in islets of diabetic donors (Dorrell et al., 2016; Dror et al., 2023; Parveen et al., 2023; Rubio-Navarro et al., 2023; Weng et al., 2023). Yet it is not clear if b-cell subsets inter-convert under diabetes-related metabolic stress due to b-cell plasticity (Zhang et al., 2022), or that b cells with better function were made at reduced proportions that are promoted by diabetes-predisposing factors. Addressing these possibilities is important, because environmental factors contribute to ~half of all human diabetes with unknown mechanisms (Laakso and Fernandes Silva, 2022).

Our previous work revealed that Neurog3^+^ islet progenitors are heterogenous in respect to their expression levels of TF Myt1, a Neurog3 target that is detected in all differentiated islet cells (Liu et al., 2019). The result is the co-presence of transient Myt1^+^Ngn3^+^ (M^+^N^+^) and Myt1^−^Ngn3^+^ (M^−^N^+^) cells. The M^+^N^+^ progenitors have higher levels of DNA methylation in a DNA enhancer of *Arx*, a key fate-specifying gene for a cells (Dhawan et al., 2011). They favor b-cell fate while the M^−^N^+^ progenitors a fate, although they both can give rise to b cells (Liu et al., 2019). Because enhancer DNA methylation is also important in b-cell maturation and identity (Dhawan et al., 2011; Dhawan et al., 2015; Liu et al., 2019; Papizan et al., 2011), we postulated that M^+^N^+^ and M^−^N^+^ progenitors give rise to b-cell subtypes with differential enhancer methylation associated with their heterogeneity in gene expression and function

## Results

### Neurog3^+^ islet progenitor subsets with differential Myt1 expression give rise to stable b-cell subtypes of different levels of proliferation, secretory function, and viability

**Figure 1:**
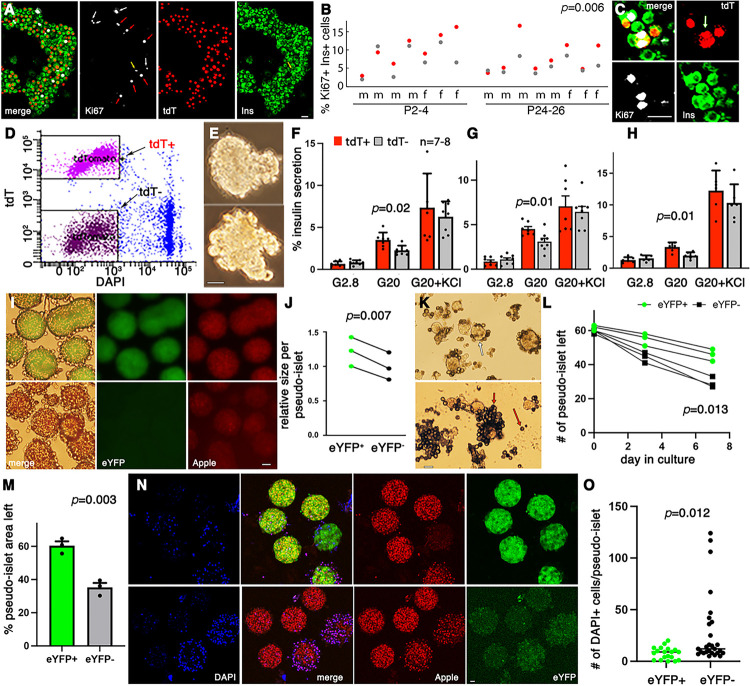
Postnatal b cells derived from M^+^N^+^ and M^−^N^+^ progenitors have different function (A-C) b-cell proliferation assays. (A) Islets were picked, dissociated, and cytospun onto slides for Ki67 staining and quantification. Ki67 signal (white) marks cycling cells; tdT (red) identifies the descendants of M^+^N^+^ progenitors; insulin (green) marks b cells. White arrows, tdT^−^Ki67^+^ cells; red arrows, tdT^+^Ki67^+^ cells; yellow arrow, a non-b-Ki67^+^ cells. (B) The % of tdT^+^ or tdT^−^ b cells expressing Ki67 in each mouse (m: males. f: females). *p* value is from paired student t-test (two-tail type 2 error). (C) A dividing tdT^+^ b cell with two separating red nuclei (arrow). (D-H) Insulin secretion assays in pseudo-islets from b-cell subsets. tdT^+^ and tdT^−^ islet cells were separated by FACS (D), made into pseudo-islets (E), and assayed for insulin secretion in response to G2.8 (2.8 mM glucose), G20, or (G20 + 30 mM KCl). The % of total insulin secreted during a 45 minutes window was presented. (F, G) Secretion from pseudo-islets of 2-months old *MNA* male or female mice, respectively. (H) Pseudo-islets secretion from 8-months old *MNA* mice, with males and females mixed. Presented are (mean + SEM). *p* values are calculated with unpaired t-test. (I-O) b-cell death assays in pseudo-islets. (I) Examples of newly-formed pseudo-islets (5 days in hanging drops) from eYFP^+^ and eYFP^−^ b cells of MNYI mice. (J) Relative pseudo-islet size (represented by pseudo-islet area measured from microscopic images). (K) Images of typical live pseudo-islet (white arrow points at one example) 7-days after culture at 11 mM glucose, stained with trypan blue to visualize dead cells (red arrows). The numbers (L) and areas (M) of the surviving pseudo-islets (white arrow) were used to compare the viability of two types of pseudo-islets. Three batches of pseudo-islets were used for this assay. (M) The proportions of islets (using area as a parameter) that survived. (N, O) Pseudo-islet cell death induced by 24-hours treatment by 20 mM glucose and 0.6 mM palmitate in eYFP^+^ and eYFP^−^ pseudo-islets. Presented in (M) are (mean + SEM), with *p* values calculated with unpaired t-test. Scale bars, 20 mm. For all assays, at least two batches of pseudo-islets were used, derived from different islet isolation, dissociation, cell sorting, and reaggregation plates.

We examined whether b cells that are derived from Myt1^+^Ngn3^+^ (M^+^N^+^) and Myt1^−^Ngn3^+^ (M^−^N^+^) progenitors have different properties. *Myt1*^*cCre*^*; Neurog3*^*nCre*^*; Ai9* (*MNA*) mice were derived, indelibly marking the descendants of M^+^N^+^ progenitors with tdTomato (tdT) (Liu et al., 2019). At neonatal and newly weaned stages [postnatal day 2 (P2) – P26], significantly higher portions of tdT^+^ b cells express Ki67 than tdT^−^ cells (Fig. 1A, B). Ki67^+^tdT^+^ cells with double nuclei were observed, consistent with them undergoing cell division (Fig. 1C).

Pseudo-islets were made from FACS-purified tdT^+^ and tdT^−^ islet cells that include all endocrine cell types and assayed for GSIS (Fig. 1D, E). Two-months old tdT^+^ pseudo-islets secrete significantly higher levels of insulin than tdT^−^ samples in response to 20 mM glucose but not to 30 mM KCl, from both male (Fig. 1F) and female (Fig. 1G) samples. Similar results were observed in 8-months-old tdT^+^ and tdT^−^ cells (Fig. 1H). In addition, pseudo-islets made from pure b cell subsets of 2-months-old *Myt1*^*cCre*^*; Neurog3*^*nCre*^*; Rosa26*^*eYFP*^*; Ins2*^*Apple*^ mice (noted as *MNYI*) showed similar results, with descendant b cells of M^+^N^+^ progenitors (eYFP^+^) having higher GISS compared to eYFP^−^ cells (Fig. S1A - C). In *MNYI* mice, the *Rosa26*^*eYFP*^ allele labels the descendants of M^+^N^+^ progenitors with eYFP production, whereas *Ins2*^*Apple*^ labels b cells with fluorescent protein Apple (Stancill et al., 2019), enabling isolation of pure b-cell subsets.

When producing pseudo-islets using MNYI b cells, we noticed that those from eYFP^−^ cells are usually smaller than from eYFP^+^ cells, although the same number of starting cells were used (Fig. 1I, J). We therefore tested if these b-cell subtypes have different viability *in vitro*. After 4 – 7 days in culture under 11 mM glucose, significantly more eYFP^+^ pseudo-islets/islet cells survive compared to eYFP^−^ samples (Fig. 1K-M). Similarly, the co-presence of high glucose and palmitate, a condition that was widely used to mimic metabolic stress in T2D, leads to more pseudo-islet cell death in eYFP^−^ than in eYFP^+^ b cells (Fig. 1N, O).

We last compared the mitochondrial activity in these b-cell subsets. The mitochondria of adult eYFP^+^ b cells have a statistically significant but only slightly higher (~6%) level of transmembrane potential (p=0.011) compared to eYFP^−^ cells (Fig. S1D, E). But they have similar levels of mitochondrial mass and ADP/ATP ratios (Fig. S1E, F). The significance of these findings are not known and not further pursued in this study.

These above results suggest that different islet progenitors give rise to stable b-cell subtypes with different ability to proliferate, function, and survive, leading us to determine the genetic/epigenetic basis for the production and activity of the two b-cell subtypes.

### Neonatal b-cell subtypes have different transcriptional profiles that cannot be attributed to asynchronous maturation

**Figure 2:**
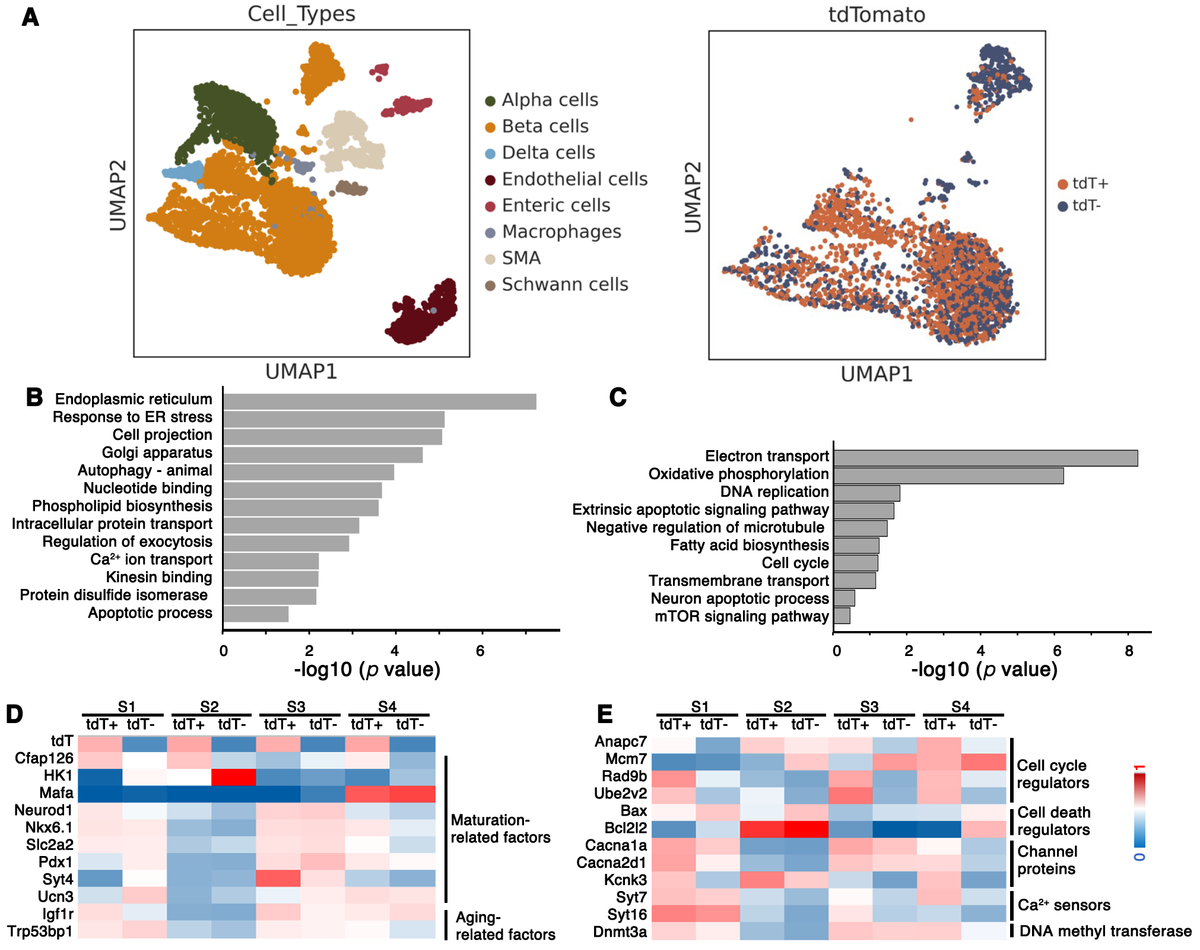
ScRNA-seq identifies DEGs in newly born b-cell subtypes. Islets from P2 *MNA* mice were isolated, dissociated, and used in inDrop-seq. The b cells were then grouped as tdT^+^ and tdT^−^ sub-populations for comparison. (A) UMAPs showing the clustering of different cell types within islets (left) and the b cell subtypes with *tdT* expression highlighted (right). (B, C) Terms that are enriched in the P2 tdT^+^ (B) and tdT^−^ (C) b-cell subtypes, analyzed using DAVID. (D, E) Heat maps showing the relative expression of several genes that are not (D) or are (E) differentially expressed in tdT^+^ and tdT^−^ b cells. Presented are log-transformed expression levels which were normalized against their mean across the four replicates. To highlight the level differences between tdT^+^ and tdT^−^ cells within each replica (S1-S4), they were presented side-by-side. Note that there are large inter-sample variations, likely reflecting the volatile nature of heterogeneity in individual mouse.

scRNA-seq was coupled with lineage tracing to compare the transcriptomes of b-cell subtypes derived from the M^+^N^+^ and M^−^N^+^ progenitors. Islet cells from P2 *MNA* mice were used for inDrops sequencing. Male and female samples were combined because no sex-based difference in b cell function has been reported at this stage. After filtering out low quality droplets and cells (Fig. S2A, B), we identified 1,275 tdT^+^ and 2,183 tdT^−^ single b-cell transcriptomes from four batches of biological replicates (Fig. 2A, Table S1). There are a total of 919 differentially expressed genes (DEGs) between these cell subtypes, including 362 down- and 557 up-regulated in the tdT^+^ cells (Table S2). Gene ontology (GO) analysis showed that the upregulated genes comprise *ER, Golgi function, Response to ER stress, Calcium ion transport*, and *Apoptosis* (Fig. 2B). Those downregulated associate with *Electron transport, Oxidative phosphorylation, DNA replication*, and *Cell cycle* (Fig. 2C). All these processes are known to regulate b-cell proliferation, function, and death, corresponding to thei detected functional differences between these b-cell subtypes.

Intriguingly, supervised analyses showed that the DEGs include neither key maturation-defining gene such as *Cfap126, HK1, Mafa, NeuroD, Nkx6.1, Pdx1, Slc2a2, Syt4,* or *Ucn3*, nor important aging markers such as *Igf1r* and *Trp53BP1* (Fig. 2D). Instead, the DEGs include cell cycle regulators (*Anapc7, Nek4*, and *Ccng1*), cell death genes (*Bax* and *Bcl2l1*),K^+^ or Ca^2+^ channel genes (*Cacna1a, Cacna2d1*, and *Kcnk3*), and vesicular Ca^2+^ sensors (*Syt7* and *Syt16*) (Fig. 2E). In addition, we found that *DNMT3a*, the *de novo* DNA methylation enzyme, has higher expression level in the tdT^+^ b cells, consistent with our report that a key difference between the M^+^N^+^ and M^−^N^+^ progenitors is their differing DNA methylation patterns (Liu et al., 2019). These findings suggest that the DEGs cannot be attributed to asynchronous maturation or aging of b-cell subsets. Rather, they likely arise from differential genetic/epigenetic program(s) in their respective progenitors, which propagate in postnatal stages to define stable b-cell subtypes. We therefore compared the expression profiles of adult tdT^+^ and tdT^−^ b cells.

### The adult b cell subtypes from M^+^N^+^ or M^−^N^+^ progenitors maintain differential gene expressions

**Figure 3:**
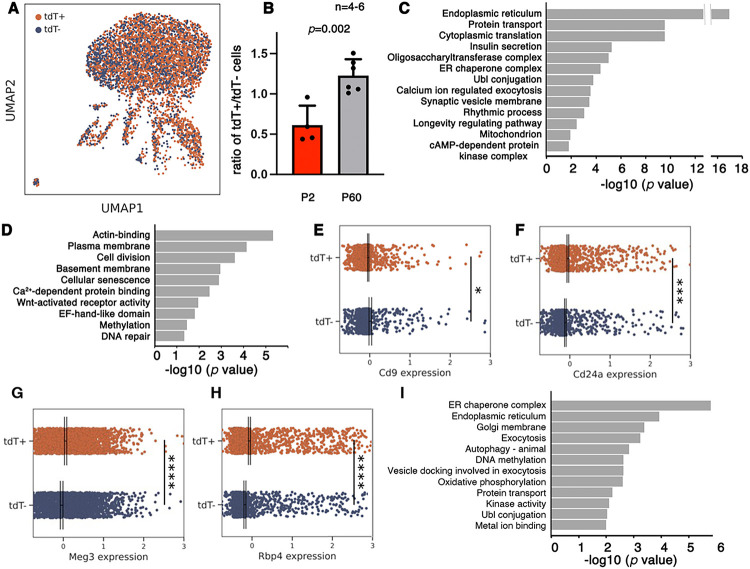
DEGs between P60 b-cell subtypes have established roles for b cells. Islets from P60 *MNA* mice were hand-picked and used for scRNA-seg. The b cells were then grouped as tdT^+^ and tdT^−^ sub-populations for comparison. (A) A UMAP showing sub-clustering of P60 b cells with tdT expression highlighted. (B) The ratio of tdT^+^/tdT^−^ cells at P2 and P60, determined via scRNA-seq. Mean + SEM were presented. *P* value was calculated using unpaired t-test. (C, D) Pathways/terms that are enriched in the tdT^+^ (C) and tdT^−^ (D) b-cell subtypes, determined using DAVID. (E-H) The expression levels of several candidate genes in b-cell subtypes (*: p<0.05. ***: p<0.001. ****: p<0.0001, determined using a Kruskal-Wallis test followed by a *post hoc* Mann-Whitney U). (I) Pathways/terms that are enriched in the DEGs shared by P2 and P60 samples.

scRNA-seq was used to compare the transcriptomes of P60 tdT^+^ and tdT^−^ b cells in *MNA* mice. The male and female samples were initially processed separately, because b-cell dimorphism has been established at this stage. We obtained 1,319 tdT^+^ and 1,164 tdT^−^ high quality single b-cell transcriptomes in three male islet samples, in addition to 1,639 tdT^+^ and 1,230 tdT^−^ b cells in three female replicas (Fig. 3A. Fig. S2C, D. Table S1). When male and female samples were analyzed separately, both showed similar pathways that are differentially expressed between the tdT^+^ and tdT^−^ b cells (Table S3). We therefore analyzed and presented results after combining the male and female samples. Note that the portion of tdT^+^ b cells is higher in P60 than P2 (Fig. 3B), consistent with the higher proliferation rate of tdT^+^ b cells in early postnatal stages (Fig. 1A-C).

The P60 tdT^+^ and tdT^−^ b cells have 1,867 DEGs, with the majority (1,614) upregulated in the tdT^+^ cells (Table S4). Consistent with the higher GSIS of tdT^+^ b cells, the down-regulated genes in these cells are enriched in several processes that inhibit GSIS, including *Actin-binding* (Kalwat and Thurmond, 2013) and *Cellular senescence* (Aguayo-Mazzucato et al., 2017; Thompson et al., 2019) (Fig. 3C); while several upregulated processes are known to promote GSIS, including *Protein translation/transport, Insulin secretion, ER chaperone complex, Mitochondrion, Calcium ion-regulated exocytosis*, and *cAMP-dependent protein kinase complex* (Fig. 3D). Notably, the DEGs include down-regulation of *CD9* and up-regulation of *CD24a, Gpx3*, and *Rbp4* (Fig. 3E-I), all have been associated with b-cell heterogeneity (Camunas-Soler et al., 2020; Dorrell et al., 2016; Dror et al., 2023; Mawla and Huising, 2019; Xin et al., 2018). More importantly, CD9^Low^ or CD24^High^ b cells were reported to have higher GSIS than CD9^High^ or CD24^Low^ cells, respectively (Dorrell et al., 2016; Dror et al., 2023).

We next examined if any of the DEGs between P2 b-cell subtypes are preferentially retained at P60. Among the 919 P2 DEGs, 193 were retained at P60 (Table S4), a 2.5-fold enrichment (p=2.37E-33, hypergeometric analysis). Most of these 193 genes are upregulated in P60 tdT^+^ b cells and they are enriched for ER/Golgi function, vesicle production/secretion, and quality controls (Fig. 3I). These findings suggest that despite the dynamic gene expression changes during postnatal b-cell maturation, a substantial number of genes maintain their differential expression between the b-cell subtypes from newly-born to adult stages. We therefore tested the functional contribution of some DEGs in b-cell subtypes.

### Several DEGs may contribute to differential GSIS in tdT^+^ and tdT^−^ b cells

**Figure 4:**
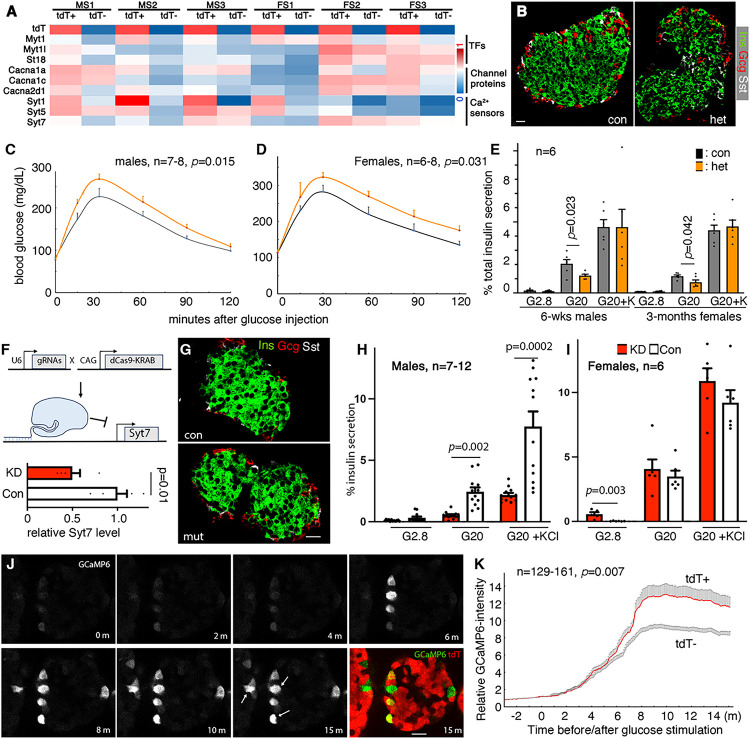
Several DEGs between P60 b-cell subtypes contribute to their different secretory function. (A) Heat maps showing the relative expression of several genes that are differentially expressed in P60 tdT^+^ and tdT^−^ b cells. Data presentation followed that in Fig. 2D, E. (B-E) The functional effects of reducing *Myt TFs* dosage in mouse islets. (B) Immunofluorescence (IF) of typical islets from 4-weeks old control (*Myt1*^*F/+*^*; Myt1L* ^*F/+*^*; St18* ^*F/+*^) and *Myt1*^*F/+*^*; Myt1L* ^*F/+*^*; St18* ^*F/+*^*; Pdx1* ^*Cre*^ (het) mice. (C, D) IPGTT results of 6-weeks old male (C) and 3-months old female mice (D). Mean + SEM were presented. *p* values were calculated using 2way ANOVA. (E) GSIS of islets from control and *Myt TF* het mice. % of insulin secretion within a 45-minutes window was presented (mean + SEM). The conditions used were: G2.8, G20, and (G20 + 30 mM KCl). *P* values were calculated using unpaired t-test. (F-I) The effects of reduced *Syt7* production in islet cells. (F) The strategy to reduce *Syt7* expression, using two gRNAs to recruit fusion repressor protein dCas9-KRAB to the promoter regions of *Syt7* for gene repression. (G) Morphology of 4-wks old islets from control and mutant mice. (H, I) GSIS of control and *Syt7*-KD islets. The % of the total insulin secreted within a 45 minutes window was measured. Mean + SEM were presented. *p* values were calculated using unpaired t-test. (J, K) Differences in glucose-induced Ca^2+^ entry in 2-months old tdT^+^ and tdT^−^ b cells. (J) A few snap shots of real-time GCaMP6 recording, which reports the level of cytoplasmic Ca^2+^. Basal glucose used was 1 mM; stimulatory glucose, added at time “0”, is 11 mM. (K) Quantification of Ca^2+^ influx in tdT^+^ and tdT^−^ b cells. The relative increase of Ca^2+^ signals were presented, normalized to that between −3 to 0 minutes before glucose administration. Cells with oversaturating GCaMP6 (arrows at 15 m) were not included for quantification. Mean + SEM were presented at each time point. *p* values were calculated using 2way ANOVA, with repeated measurement. Scale bars, 20 mm.

For functional tests, we chose the families of *Myt TF* (*Myt1, Myt1L*, and *St18*), synaptotagmins, and voltage-gated calcium channels (*Cacna1a, Cacna1c*, and *Cacna2d1*). Multiple members in each of these families display upregulation in the more functional P60 b-cell subtype (Fig. 4A, Table S4). Their function in GSIS has been established via gene inactivation, but not by partial loss-of function (Camunas-Soler et al., 2020; Dolai et al., 2016; Gauthier and Wollheim, 2008; Hu et al., 2020; Huang et al., 2018; Iezzi et al., 2004; Ofori et al., 2022; Thompson and Satin, 2021; Wu et al., 2015).

*Myt1*^*F/+*^*; Myt1l* ^*F/+*^*; St18* ^*F/+*^*; Pdx1*^*Cre*^ mice were derived. This reduces the levels of Myt1, Myt1L, and ST18 proteins by 34–63% in islets compared with controls (Fig. S3B, C). These levels of reduction did not impact the overall morphology of islets (Fig. 4B), but compromised glucose clearance in both male and female mice (Fig. 4C, D), accompanied by reduced islet GSIS (Fig. 4E). Note that mutant males displayed glucose intolerance earlier than females, results that usually occur when islet function was partially compromised (Gannon et al., 2018).

To test the effect of reduced *Syts* expression in b-cells, CRISPRi was used to reduce the level of *Syt7* by ~50% in mouse islets (Fig. 4F). This was achieved using two *Syt7* guide RNAs to recruit a fusion protein of dCas9 with transcriptional repressor domain KRAB to the transcription starting sites of *Syt7* (Fig. S3A) (Jensen et al., 2021). Reduced *Syt7* expression did not alter the islet morphology (Fig. 4G) but compromised GSIS in male islets (Fig. 4H). Note that this level of *Syt7* reduction did not compromise GSIS in female islets (Fig. 4I), consistent with the findings that male b cells are more liable for dysfunction (Gannon et al., 2018).

For testing the contribution of Ca^2+^ channels, we directly compared glucose-induced Ca^2+^ influx in tdT^+^ and tdT^−^ b cells in real time. The cytoplasmic Ca^2+^ influx in response to 11 mM glucose, assayed with b-cell specific GCaMP6 as a surrogate (Fig. S3B), is more robust in the tdT^+^ b cells than in tdT^−^ cells (Fig. 4J, K, S3C, Movie S1). These results, combined with known roles of voltage-gated channels in Ca^2+^ influx/secretion coupling and the identical ADP/ATP ratio in the two b-cell subtypes (Fig. S1F), suggest that higher Ca^2+^ channel expression in tdT^+^ cells contributes to their higher GSIS as well. We conclude that, multiple factors, by virtue of their differential expression, contribute to the different GSIS of b-cell subtypes. We next explored the mechanisms that establish this gene expression heterogeneity in b cells.

### The neonatal b-cell subtypes from M^+^N^+^ or M^−^N^+^ progenitors display methylation differences at a subset of putative enhancers

**Figure 5:**
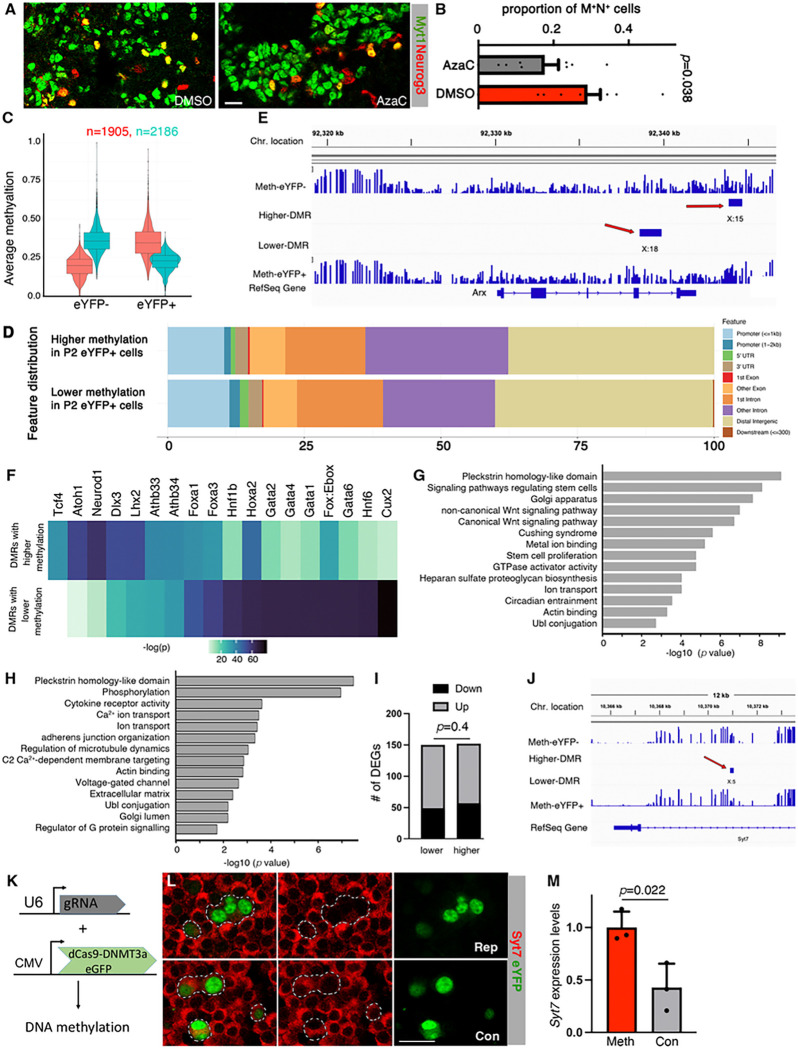
M^+^N^+^ and M^−^N^+^ progenitor-derived b cells have differentially methylated regions that may regulate gene expression. (A, B) Myt1 and Neurog3 co-expression in pancreatic buds cultured *in vitro* with or without DNMT inhibition by AzaC. Only co-staining was shown. *P* value is from t-test, two-tail type 2 errors. Scale bar, 20 mm. (C) Methylation scores of DMRs between P2 eYFP^+^ and eYFP^−^ b-cell subtypes. (D) The locations of DMRs in respect to their predicted target genes. (E) DMRs near the *Arx* locus. Shown are four tracks (from top to bottom) of CpG methylation of eYFP^−^ b cells, DMRs with higher, DMRs with lower levels of methylation in eYFP^+^ cells, and CpG methylation of eYFP^+^ b cells. Among the two DMRs (red arrows), one has higher and the other lower levels of methylation in eYFP^+^ cells. The number following “X:” indicates the number of CpG dinucleotides within the DMR. (F) DNA motifs that are enriched in the DMRs between b-cell subtypes. The motifs in DMRs with lower or higher methylation were presented separately. (G, H) Pathways/terms that are enriched in the DMR-associated genes, with either lower- (E) or higher levels of (F) methylation. (I) P2 DEGs (down- or up-regulated) that are associated with P2 DMRs (with either lower or higher levels of methylation in eYFP^+^ cells). P values was calculated using Fisher exact test, comparing with random association with all detectable genes in b cells. (J) A DMR in the first intron of *Syt7*. The annotation is identical to that in panel (B). (K-M) The effects of site-specific methylation increase near the *Syt7* locus. An U6 promoter was used to drive the expression of a guide RNA, which was expected to bring fusion protein dCas9-DNMT3a (reported by eGFP expression) to methylate DNA close by (I, J) (Liu et al., 2019). The co-expression of the two constructs in MIN6 cells (rep, or repression) resulted in significant reduction in *Syt7* mRNA (K) than controls (con, without guide RNA). Scale bar, 20 mm. Presented in K are real-time RT-PCR assays of three samples (Mean + SEM), with *p* values calculated with unpaired t-test.

Work from our lab and others has shown that cell-type specific transcriptional enhancers are selectively hypomethylated during progenitor differentiation (Barnett et al., 2020). Many hypomethylated regions (HMRs) are maintained in subsequent developmental stages, resulting in patterns that record the histories of cell fate decisions (Scott et al., 2023). Corresponding to these findings, we now detected higher levels of *DNMT3a* in tdT^+^ b cells at both P2 and P60 (Tables S2 and S4). In addition, acute inhibition of DNMTs in embryonic pancreatic buds significantly reduced the proportions of M^+^N^+^ progenitors (Fig. 5A, B). Thus, we postulated that differential enhancer methylation in islet progenitors drive cell-subtype production and maintenance.

Whole genome bisulfite sequencing (WGBS) was done on sorted eYFP^+^ and eYFP^−^ b cells from P2 *MNYI* males and female islets. This identified 78,634 HMRs, falling into 6 clusters based on their methylation level changes between the cell subtypes (Fig. S4A). Further analysis defined 4,091 differentially methylated regions (DMRs) by gating those with a minimum length of 50 bp and 2 differentially methylated CpG sites (*p*<0.05). Among these DMRs, 1,905 have lower levels of methylation in eYFP^−^ cells and 2,186 lower in eYFP^+^ cells (Fig. 5C. Table S5). Each DMR contains 2–15 differentially methylated CpGs, spreading up to 3kb (Fig. S4B, C). Roughly 60% of the DMRs localize within the putative enhancer regions (from 2 kb upstream of the transcription starting site to the 3’ UTR, Fig. 5D), while 80% within 100 kb, of their putative target genes (Fig. S4D). Note that the mean-difference in methylation of these DMRs is ~1.6-folds, lower than the usually larger (>2-folds) differences observed between diverse cell types and developmental timepoints (Fig. 5C) (Barnett et al., 2020; Liu et al., 2019; Scott et al., 2023). This is expected because of the higher levels of similarity between b subtypes. Also note that we detected DMRs in the Arx locus in these subtypes (Fig. 5E), which is differentially methylated in the M^+^N^+^ and M^−^N^+^ islet progenitors (Liu et al., 2019). This result is consistent with our hypothesis that the differential DNA methylation patterns are passed down do differentiated islet cells.

The P2 DMRs are enriched for binding motifs of several TFs belonging to the FOXA, GATA, and HNF families (Fig. 5F), all of which have established roles in b-cell production and/or function (Golson et al., 2015; Gu et al., 2010; Gupta et al., 2007; Lorberbaum and Sussel, 2017; Reizel et al., 2021; Weng et al., 2023). The implication is that b-cell subtypes may express same levels of these TFs (Table S2), but they can transcribe different levels of their target genes due to the presence of these DMRs, leading to differential function.Supporting this possibility, genes associated with P2 DMRs (3,143 and 2,871 corresponding to lower and higher levels of methylation in eYFP^+^ cells, respectively, Table S5) are enriched for pathways that regulate *Golgi, Wnt Signaling, Ion transport, Circadian entrainment, Ca*^*2+*^ *transport*, and *Actin binding* (Fig. 5G, H). Note that 902 genes are associated with DMRs with lower methylation while also with DMRs higher methylation in eYFP^+^ cells (Table S5). This may suggest some coordinated regulation of these genes by multiple gene regulatory elements.

### The DMRs between P2 b-cell subtypes associate with DEGs of both neonatal and adult b-cell subtypes

We next asked if any P2 DMR-associated genes are differentially expressed between the two b-cell subtypes at P2 and P60. Among the 919 DEGs at P2, 150 and 152 have DMRs with lower or higher levels of methylation in eYFP^+^ cells, respectively (Table S2), significant enrichments over non-differentially expressed genes (*p*=0.0004, Fisher Exact test). The ratios of down- and up-regulated genes are similar in those associated with lower- or higher-methylated DMRs (Fig. 5I, *p*=0.4. Fisher Exact test), consistent with the idea that changes in enhancer DNA methylation can be both activating and repressing (Dhar et al., 2021). Corroborating this conclusion, ectopic methylation of a DMR in the first intron of *Syt7* (Fig. 5J) using a proven dCas9-DNMT3a construct (Liu et al., 2019) significantly reduces *Syt7* expression in a MIN6 b-cell line (Fig. 5K, M). Note that this DMR was chosen because *Syt7* expression levels can impact b-cell function and the methylation level in this DMR changes during b-cell aging, implying its functional significance (Avrahami et al., 2015).

Similar to P2 gene expression, 566 of the 1,867 P60 DEGs are associated with the DMRs at P2 (Table S4. *P*<0.0001, Fisher Exact test). These data support an idea that DMRs at P2 can impact the gene expression at later stages, likely by maintaining their DNA methylation landscape and/or promoting stable gene networks. We therefore tested how DNA methylomes evolve in the two b-cell subtypes from newly-born to adult stages.

### DNA methylomes in postnatal b cells are highly dynamic but adult b-cell subtypes maintain DMRs that associate with their DEGs

**Figure 6:**
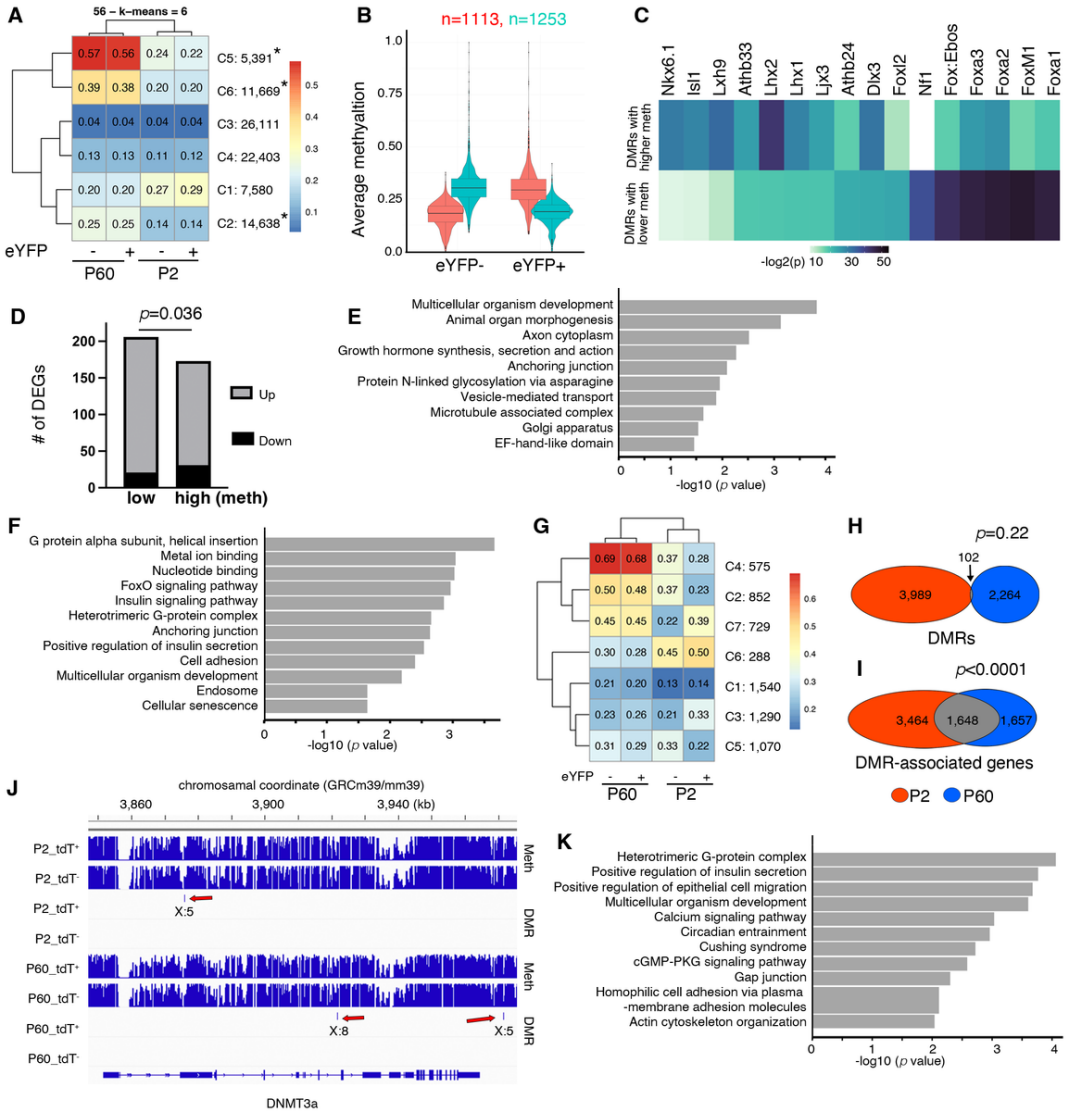
DNA methylome in postnatal b cells is dynamic but adult b-cell subtypes have DMRs that associate with a common set of genes as those at P2. Genome-wide methylome analysis followed that in Fig. 5. (A) Clustering of HMRs in P2 and P60 b cells based on their levels of DNA methylation. The number of HMRs in each of the six clusters (C1 to C6) are shown. “*” indicate the clusters with >1.8 folds difference in their mean. (B) Methylation scores of DMRs between the P60 b-cell subtypes. (C) Enriched motifs in the DMRs with lower or higher levels of methylation (meth) in eYFP^+^ b cell subtypes. (D) DMR-associated DEGs between the P60 b-cell subtypes. *P* values is from Fisher exact test. (E, F) Terms that are enriched for DMR-associated DEGs, with lower (E) or higher (F) levels methylation in eYFP^+^ cells. (G-K) DMR/DEG overlaps between P2 and P60 cell subtypes. (G) Clustering of all DMRs at P2 and P60. (H) The number of DMRs that is retained in P2 and P60 cell subtypes. (I) The overlap between DMR-associated genes at P2 and P60. P values were calculated using hypergeometric analysis. (J) An example (*DNMT3a*) to show that a gene can be regulated by DMRs detected at P2 and P60, although the locations of the DMRs may shift (red arrows). (K) Pathways that are enriched in the P60 DEGs that also associated with P2 and P60 DMRs.

WGBS in P60 b-cell subtypes identified 68,485 HMRs (Fig. S5A), 59,327 of which overlap with that at P2. They constitute 6 clusters, with three (a total of 31,698 HMRs) showing >1.8 fold methylation changes between P2 and P60 total-b cells (Fig. 6A), underscoring the dynamic DNA methylation changes from neonatal to adult stages. In contrast, the P60 b-cell subtypes have relatively small DNA methylation differences, with 1,113 and 1,253 DMRs that have higher or lower methylation in eYFP^+^ b cells compared to eYFP^−^ cells, respectively (Fig. 6B). These DMRs have 2–14 CpG dinucleotides differentially methylated, spanning 50 bp to 3.5 kb (Fig. S5-B, C), with the majority (>70%) locate in the putative regulatory regions of their predicted target genes (Fig. S5D).

Similar to that of P2, the P60 DMRs are enriched for motifs of several b-cell regulators of function/proliferation, including *Nkx6.1, Isl1, Foxa TFs, and FoxM1* (Fig. 6C). These DMRs associate with 1,968 and 1,735 genes, respectively (Table S6), which are enriched for *Actin binding, Calcium ion transport, Golgi, Microtubule, Rhythmic process*, and *Wnt signaling* (Fig. S5E, F).

There are 206 and 173 DEGs between P60 b-cell subtypes that are associated with P60 DMRs with lower or higher levels of methylation in eYFP^+^ cells, respectively (Table S4 and Fig. 6D), a significant enrichment (*p*<0.0001, Fisher Exact test). At this stage, significantly more up-regulated DEGs are associated with DMRs with lower levels of methylation (*p*=0.036, Fisher exact test. Fig. 6D). The DEGs that are associated with lower levels of DNA methylation are enriched for *Organism development, Organ morphogenesis, Growth hormone synthesis, secretion, and action, Anchoring junction, Vesicle-mediated transport, Microtubule associated complex, and etc*. (Fig. 6E); while those with higher levels of methylation are enriched for *Metal ion binding, FoxO signaling, Insulin signaling, G-protein, Positive regulation of insulin secretion, Cellular senescence, and etc*. (Fig. 6F). These findings suggest that differential methylation at a subset of putative enhancers distinguish b-cell subtypes at P60. We therefore examined whether any DMRs are maintained between b-cell subtypes from P2 to P60 to account for their stability.

### A significant portion of genes that are associated with P2 DMRs between b-cell subtypes also associate with those at P60

Hierarchical analysis did not show any cluster that are differentially methylated at both stages (Fig. 6G). Only 102 DMRs remain unchanged (Fig. 6H), representing no enrichment (*p*=0.22, hypergeometric test). However, about one third (1,648) of the DMR-associated genes overlaps between P2 and P60 (Fig. 6I. Table S6), a 2.2-folds enrichment (*p*=1.11E-295, hypergeometric test). In other words, putative enhancers of 1,648 genes, e.g., *DNMT3a*, are differentially methylated between the b-cell subtypes at both P2 and P60, although the exact locations of the DMRs have shifted (Fig. 6J). In addition, 193 of the 1,648 common DMR-associated genes display differential expression at P60, a significant overlap (*p*=0.0001, Fisher exact test. Table S6). They are enriched for GSIS-related processes such as *Heterotrimeric G-protein complex, Positive regulation of insulin secretion, Calcium signaling, Circadian entrainment, Actin cytoskeleton organization*, andetc.(Fig. 6K).

Thus, our combined findings so far, together with the known roles of DNA methylation in b cells, suggest that differential enhancer methylation in early progenitor subsets can propagate after birth, producing stable b-cell subtypes at adult stages. This conclusion sets the stage to test if any factor modulates the risk of diabetes by changing the proportions of b-cell subtypes.

### Maternal overnutrition leads to reduced *DNMT3a* expression in and reduced proportion of M^+^N^+^ progenitor cells

We tested whether maternal overnutrition can alter the proportions of M^+^N^+^ and M^−^N^+^ islet progenitor cells. Maternal nutrition is a prominent risk factor for diabetes in offspring (Friedman, 2018), supporting a theory known as Developmental Origin of Health and Disease (DOHaD) in human diabetes. This theory posits that maternal factors program b cells to certain levels of activities that maybe unfit for postnatal GSIS when nutrient levels change (Wadhwa et al., 2009). Yet the exact cellular and molecular mechanism remains unclear (Elsakr et al., 2019).

**Figure 7:**
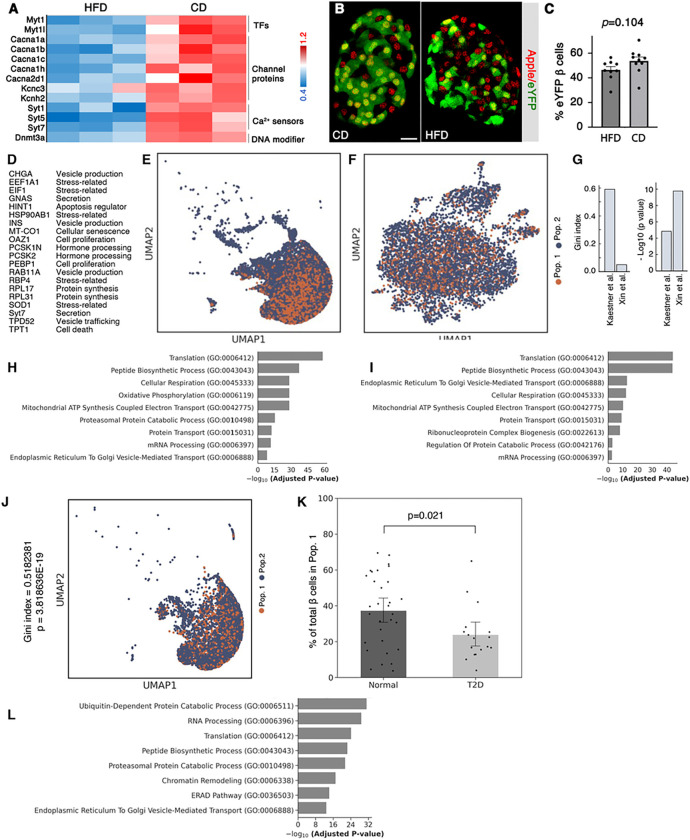
A reduction of b-cell subtype of higher function corresponds to diabetes development in mice and human. (A) A few examples of genes with changed expression in Neurog3^+^ cells with HFD or control diet (CD) treatment. Shown are the levels of expression relative to that in CD-treated cells. (B, C) Examples and quantification of labeled b cells in P4 *MNYI* mice after maternal HFD/CD treatment. Bar = 20 mm. *p* value is from t-test (two-tail type 2). (D) The 20 mouse DEGs selected for human b-cell subpopulation studies. See Table S8 for references that helped with their selection. (E, F) UMAPs of human b-cell populations based on the aggregated expression of the 20 genes listed in (A), using available data in Kaestner et al. (Kaestner et al., 2019) and Xin et al. (Xin et al., 2018). (G) Gini indices and respective *p*-values to measure if the distribution of 20 genes was equal amongst all b cells. The null hypothesis is that the distribution was equal. (H, I) Processes that are enriched in Pop. 1 cells of Kaestner et al. (E) or Xin et al. (F). The top 9 processes are presented. (J, K) Sub-clustering and quantification of b-cell subpopulations from T2D donors. The T2D b cells from Kaestner et al. (Kaestner et al., 2019) were used. Presented are (Mean ±SEM). *P* value is calculated using t-test. (L) Pathways that enriched in Pop. 1 T2D donor islets.

We compared the gene expression in E15.5 Neurog3^+^ islet progenitors that are maternally exposed to control and high-fat-diet (CD and HFD) (Fig. S6). HFD treatment results in downregulation of 2,155 genes and upregulation of 1,414 in Neurog3^+^ cells (Table S7). The downregulated genes are enriched for processes including *Ubl conjugation pathways, microtubules, Ca*^*2+*^ *transport, ER, Golgi, chromatin regulators*, and *Ca*^*2+*^*-regulated exocytosis* (Fig. S6B), similar to that of P2 tdT^+^ cell-enriched terms (Fig. 3C). The upregulated genes are enriched for *protein translation, mitochondrion, unfolded protein response*, and several biosynthetic/metabolism pathways (Fig. S6C). Intriguingly, maternal HFD reduces the expression levels of *Myt1*, several channels, Ca^2+^ sensors, and *DNMT3a* in Neurog3^+^ progenitors (Fig. 7A), genes that are enriched in P2 and P60 b-cell subtypes with better function (Fig. 2E). Corresponding to these results, there is a trend of reduced M^+^N^+^ progenitor-derived b-cell subtype in perinatal *MNYI* mice exposed to maternal HFD (Fig. 7B, C). This weak phenotype likely reflects the reduced b-cell subtype with better function (corresponding to the reduced *Myt1* expression in *Neurog3*^*+*^ cells), which is mitigated by compensatory proliferation of these cells in response to higher nutrient levels (Mosser et al., 2015). Unfortunately, because b cells re-activate *Neurog3* under metabolic stress during diabetes development (Talchai et al., 2012), which we expect to label M^−^N^+^ progenitor-derived b cells after birth, we could not compare the function of the adult b-cell subtypes from M^−^N^+^ and M^−^N^+^ progenitors in the maternal HFD model. Therefore, we evaluated if the equivalent b-cell subtypes exist in human islets and if their proportion changes under diabetic conditions.

### DEGs from mouse b cell subtypes subdivide human b cells into distinguishable populations.

Currently, there is a lack of consensus markers to reproducibly subdivide human b cells across all studies, likely due to variable genetic/epigenetic predisposition, asynchronous maturation, proliferation, or physiological stimuli (Mawla and Huising, 2019). We reasoned that a combination of multiple markers can overcome these sources of variation and increase the reliability with which we can identify human b-cell subpopulations. We therefore selected 20 mouse DEGs as a gene signature for this goal (Fig. 7D), based on their known function and on their relatively high levels of expression in human b cells (Table S4) (Blodgett et al., 2015).

The gene signature were applied to two available single-cell transcriptome datasets, one includes all control b cells from the Human Pancreas Analysis Program (HPAP) (Kaestner et al., 2019) and the second from a cohort of 12 non-diabetic patients (Xin et al., 2018). Our gene signature consistently classify both b-cell datasets into visually notable subpopulations – population 1 (Pop. 1) and Pop. 2 (Fig. 7E, F). Gini index measurement verified this observation, showing that the signature genes were expressed in a clustered manner (Fig. 7G) (O’Hagan et al., 2018). Pre-ranked GSEA revealed strikingly consistent pathways that are enriched in Pop. 1 of both datasets as those activated in the adult mouse tdT^+^ b cell subtype, which include protein/peptide translation, trafficking, and mitochondria-associated pathways (Fig. 7H, I).

To explore if the proportions of Pop. 1 and Pop. 2 change in dysfunctional T2D human islets, we examined their relative representation in the T2D data set of (Kaestner et al., 2019). Our gene signature was highly effective in classifying disease state b cells into Pop. 1 and Pop. 2 b cells as in control samples using Gini index analysis (Fig. 7J). More importantly, we found a decline in Pop. 1 b cells (corresponding to the better function mouse b-cell subtype) in the T2D samples as compared to the controls (Fig. 7K). Given the known reduction of functional secretion and insulin-producing b cells in T2D, a decrease in this population associated with these same features is expected. Pre-ranked GSEA further corroborates this with Pop. 1 b cells in the T2D population having functional changes, with the upregulation of catabolic processes, a noted side-effect of T2D disease (Zanetti et al., 2020) (Fig. 7L).

## Discussion

We show here that embryonic islet progenitors with differential gene expression and DNA methylation give rise to b-cell subtypes of different function in postnatal mice. Maternal overnutrition, a diabetes risk factor, alters the proportion of these progenitors. Human donor islets contain equivalent b-cell subsets, with the one of higher functionality reduced in diabetic donors. Thus, deregulating the proportions of b-cell subtypes during early embryogenesis could predispose the risk of diabetes. The implication is that manipulating DNA methylation in islet progenitors, e.g. with food supplements during gestation (Li et al., 2014), may reduce the risk of diabetes. In addition, controlling nutrient levels in culture could aid the production of functional b-like cells for transplantation-based diabetes therapy. Broadly speaking, we suggest that progenitor heterogeneity may underly the presence of cell subtypes in many other organs, which can be explored with our refined lineage tracing.

By far, most studies on b-cell heterogeneity rely on sampling single b cells at particular stages. These led to a popular conclusion that most observed heterogeneity results from unstable cell states (Bonner-Weir and Aguayo-Mazzucato, 2016; Xin et al., 2018; Zeng et al., 2017). Some lineage-marking studies support this conclusion. For example, Bader et al. detected two b-cell subsets that express different levels of *Fltp/CFAP126* in mice, but one of them convert to the other over time (Bader et al., 2016). In contrast, another lineage study showed the presence of virgin b cells that stay immature for nearly half the lifespan in mice (Lee et al., 2021). Along a similar line, two recent studies, using DNA/histone methylation as lineage-surrogates, concluded that stable b-cell subtypes exist (Dror et al., 2023; Parveen et al., 2023). However, the virgin b cells represent only a few percent of all b cells, while DNA/histone methylation patterns change according to physiological conditions [our results here and (Avrahami and Kaestner, 2019; Avrahami et al., 2015; Hall et al., 2018; Hall et al., 2019; Wortham et al., 2023)]. Thus, the stability of b-cell subtypes needs direct substantiation, a prerequisite for determining the stage when subtypes arise and whether/how deregulating proportions of cell subtypes causes diabetes.

Our combinatorial lineage tracing enables longitudinal study of roughly half of all b cells that are derived from an islet progenitor subset. We clearly demonstrated the stability of functionally/genetically/epigenetically distinct b-cell subtypes. We further verified several cell markers that were previously shown to divide rodent and human b cells into functionally distinct subtypes (Dorrell et al., 2016; Dror et al., 2023), including cell surface markers CD9 and CD24 that can be used for live cell subtype purification. We also identified several DEGs (*Myt* TFs, *Syt* genes, and Ca^2+^ channels) that may contribute to the functional differences of these cell subtype, underscoring the multifactorial nature of b-cell heterogeneity.

For mechanistic understanding, we suggest that differential DNA methylation in islet progenitors is an important regulator of b-cell subtype production and risk of diabetes. First, inhibiting *DNMT* activity reduces the portion of the M^+^N^+^ progenitors in embryonic pancreas. Second, *DNMT3a* is detected at higher levels in the b-cell descendants of M^+^N^+^ cells (with higher levels of GSIS) compared with that of M^−^N^+^ cells. Third, the thousands DMRs between b-cell subtypes are enriched for motifs recognized by TFs of the *GATA, FoxA, FoxM1*, and the *HNF* TF families, which facilitate b-cell production, function, and proliferation. In addition, these DMRs preferentially associate with DEGs between the two b-cell subtypes, implying their functiaonla significance. Fourth, the Neurog3^+^ progenitors that are exposed to maternal overnutrition, an established risk factor for diabetes, express lower levels of *DNMT3a* and *Myt1*. In other word, maternal overnutrition reduces the proportion of M^+^N^+^ progenitors, likely resulting in a reduction of better-function b-cell subtype and a higher risk of diabetes in offspring. Consistent with this latter possibility, we show that donor islets from diabetic patients have lower proportion of the b-cell subset that is equivalent to the M^+^N^+^ progenitor-derived cells.

Our studies left several pressing questions unanswered. First, it is unclear if each of our identified b-cell subtypes contain sub-subtypes. For example, we show that the tdT^+^ b cells (with higher secretory function) express higher levels of *Rbp4*. But its expression was reported to anti-correlate with exocytotic activities (Camunas-Soler et al., 2020). In addition, we did not detect differential expression of CD63, which marks b cells of higher GSIS (Rubio-Navarro et al., 2023). Neither did we detect higher levels of *mKi67* transcripts in the tdT^+^ b cells. One explanation for these discrepancies is that the tdT^+^ and tdT^−^ b cells can be further subdivided, leading to varied expression levels in different cell sub-subtypes. Second, why M^+^N^+^ and M^−^N^+^ b-cell progenitors have different DNMT3a/DNA methylation and how postnatal b cells regulate their methylome are not known. Future studies examining how methylating (DNMTs) and demethylating (TETs) enzymes are targeted to specific loci will be informative. Lastly, our combinatorial lineage marking is not inducible. Reactivation of *Neurog3*, which happens when b cells are under stress (Talchai et al., 2012), will label M^−^N^+^ progenitor-derived b cells. Thus, although *Myt1* co-expression with *Neurog3* during embryogenesis represents the progenitors of better-function cells, this model is not fit for studying b-cell subtype stability in diabetes-prone models at postnatal stages.

## Materials and Methods

### Animal models and use

Mouse production and usage followed protocol approved by the Vanderbilt IACUC for Dr. Gu, in compliance with the policies of AALAC. Wild type CD1 mice were purchased from Charles River. Routine crosses were used derive mice of desired genotypes, determined by gene specific PCR reactions. *Neurog3*^*eGFP*^*, Neurog3*^*nCre*^*, Myt1*^*cCre*^*, Ai9* (*Rosa26*^*tdTomato/+*^), *Rosa26*^*eYFP*^*, Pdx1*^*Cre*^*, Myt1*^*F*^*, Myt1L*^*F*^, and *St18*^*F*^ allele was previously described (Hu et al., 2020). C56BL/6J.dCas9-KRAB mice (#030000) were obtained from Jax Laboratories. Transgenic *Syt7-gRNA* mice (*C57BL/6J.Tn*^*(pb−PGK1−EGFP,RNU6−gRNA:Syt7)1Ggu*^, MGI 7550749) were derived via pronuclear by the Vanderbilt Genome Editing Resource. The co-injected plasmids are *pEF1a-HA-m7pB* [(a *piggyBac* expression plasmid (Doherty et al., 2012)], another assembled by Vectorbuilder.com [containing *piggyBac* dual inverted terminal repeat sequences, two U6 promotor driven small guide RNAs (5’-AGAGAGTGTGTGCGCGCCCG and 5’-AGCGGCGGCAGAGAAGCGCG) that target the *Syt7* transcriptional start site], and an hPGK-eGFP expression cassette.

### Islet β cell preparation

Islets were hand-picked after the pancreata were digested with 0.5 mg/ml type IV collagenase (dissolved in HBSS with Ca^2+^/Mg^2+^) as previously described (Huang et al., 2018). Briefly, neonatal pancreata (younger than 1 week old) were dissected and cut into 4–6 pieces and digested with collagenase Type IV for ~ 10 minutes at 37 °C. The pancreata were broken into pieces with pipetting and washed 4 times with complete RPMI1640 media (with 11 mM glucose, 1X Penicilin/Streptomycin, and 10% FBS). Islets were then picked under a dissecting scope. Islets isolation from older mice used similar method except pancreata were perfused with the collagenase via pancreatic duct.

For islet cell immunostaining, islets were washed 3-times (3–5 minutes each) with Ca^2+^/Mg^2+^-free HBSS and partially dissociated (at 37 °C, with 0.25% trypsin, usually 3 minutes). The cells were washed 2X with complete RPMI1640 media and cyto-spun onto glass slides, fixed, and stained for protein marker expression.

For single cell-preparation (used for scRNA-seq and FACS), islets were washed 3-times (3–5 minutes each) with Ca^2+^/Mg^2+^-free HBSS. They were then dissociated to single cells (at 37 °C, with 0.25% trypsin, usually 4–6 minutes), washed 2X with complete RPMI1066 media, and resuspended for encapsulation-based scRNA-seq or for FACS-based cell isolation using BD FACSAriaTMIII. Note that for FACS, DAPI or propidium iodide was used for counter-staining to exclude dead cells. Sorted live cells were immediately used for down-stream experiments. Also note that when making MNA pseudo-islets, the collected cell population was gated to minimize including non-endocrine cells in islets, which are usually smaller than endocrine cells.

### Islet β cell proliferation, pseudo-islet production, and secretion assays

To assay for β-cell proliferation, islets from each *MNA* animal were processed as one sample, cyto-spun onto 2–4 slides for staining. After Immunoflurescence (IF) staining for Ki67 and insulin (usually with 2 slides), confocal images were taken to count the portion of Ki67^+^ β-cell subtypes. A Leica TCS-SP5 scanning or an Olympus FV-1000 confocal microscope were used for image capture.

Pseudo-islets preparation followed published protocols, with some modification (Friedlander et al., 2021). In this case, sorted cells were resuspended in enriched complete RPMI media (15% FBS) at 30,000 cells per ml. Then 30 microliters were dropped on the cover of 10 cm plates, which was inverted to cover plates with ~ 5 ml 1X HBSS. The plates were left at 37 °C in an incubator for 4 days un-disturbed. Pseudo-islets were then collected, left in fresh complete RPMI 1066 media for another day for recovery. Afterwards, pseudo-islets were used for GSIS assays followed established protocol for while islet [see below and (Hu et al., 2020)]. For viability assays, they were cultured with different levels of glucose and/or palmitate (see Fig. 1-related text). They were then assayed for viability via DAPI or trypan-blue intact or size.

### Mitochondrial activity assays

For comparing mitochondrial mass and activity, islets from 2–3 month-old *MNYI* mice (both males and females) were isolated and dissociated into single cells. They were then stained with MitoView^™^ 633 or MitoView^™^ 650 (Biotium) at 37 °C for 15 minutes in the presence of 20 mM glucose, followed by Flow-cytometry analysis (Ernst et al., 2023). These dyes assay the mitochondrial transmembrane potential and mitochondrial mass, respectively. The β-cells were gated into eYFP^+^ and eYFP^−^ β-cell subtypes in order to compare their MitoView^™^ signals. For ADP/ATP ratio in purified β-cell subtypes, an EnzyLight^™^ ADP/ATP ratio Assay Kit (BioAssay Syetems) was used, following the manufacturer’s protocols.

### Insulin secretion assays

For insulin secretion using pseudo-islets or intact islets, the % of total insulin secreted within a 45-minute window was measured unless noted. (Pseudo-)islets were allowed to recover in RPMI-FBS for 2 hours or overnight. Islets were washed twice with pre-warmed KRB solution (2.8 mM glucose, 102 mM NaCl, 5 mM KCl, 1.2 mM MgCl_2_, 2.7 mM CaCl_2_, 20 mM HEPES, 5 mM NaHCO_3_, and 10 mg/ml BSA, pH 7.4) and then incubated in KRB (37 °C) for one hour, washed with pre-warmed KRB once more. 10–15 (pseudo-)islets were then transferred into each of the wells of 12-well plates with 0.2 (pseudo-iselts) 1 ml (islets) pre-warmed KRB to start the secretion assays. The basal solution contains 2.8mM glucose in KRB. The stimulatory conditions use 20 mM glucose or (20 mM glucose + 30 mM KCl). For all assays, four or more mice of each genotype were used for islet isolation, with islets from two or more mice mixed and examined as 2–3 technical replicas. If Pseudo-islets were used, a starting volume of 200 microlitter with 20–30 pseudo-iselts was used due to their small size. Total insulin was assayed after ethanol-acid extraction as in (Huang et al., 2018). Insulin was measured with an Elisa kit from ALPCO. Assays from human islets use similar method, except the basal glucose was 3.3 mM.

### Intraperitoneal glucose tolerance test (IPGTT) in mice and IF assays in tissue sections

IPGTT followed previously described method (Hu et al., 2020). Briefly, mice were fasted overnight. Glucose was injected at 2mg/kg. Blood glucose was then measured via tail nip. IF assays follow routine method using both frozen and paraffin sections, using antibodies listed in the reagent table.

### Ca^2+^ recording with GCaM6

Ca^2+^ influx in individual β cells were monitored using GCaMP6 as a surrogate using a similar approach as in (Dickerson et al., 2019). Briefly, islets were isolated from 2-months old MNA mice. They were allowed to attach to fibronectin-coated plates with glass-bottoms overnight in RPMI1640 media. The islets were then infected with adenoviruses that express GCaMP6 specifically in β cells. Three days later, the islets were incubated (20 minutes, 37°C) in KRB supplemented with 1 mM glucose for acclimation. Real-time fluorescence imaging then followed to record was then performed using a Nikon Eclipse TE2000-U microscope equipped with an epifluorescence illuminator (Sutter, Inc), a CCD camera (HQ2; Photometrics Inc), and Nikon Elements software. Islets were perifused at 37°C at a flow of 2 mL/min with appropriate KRB-based solutions that contained glucose concentrations and compounds specified in the figures. The ratios of emitted fluorescence intensities at excitation wavelengths of 340 and 380 nm (F340/F380) were determined every 5 seconds with aid of agarose. For insulin secretion, the % of total insulin secreted within a 45-minute window was measured unless noted. (Pseudo-)islets were allowed to recover in RPMI-FBS for 2 hours or overnight. Islets were washed twice with pre-warmed KRB solution (2.8 mM glucose, 102 mM NaCl, 5 mM KCl, 1.2 mM MgCl_2_, 2.7 mM CaCl_2_, 20 mM HEPES, 5 mM NaHCO_3_, and 10 mg/ml BSA, pH 7.4) and then incubated in KRB (37 °C) for one hour, washed with pre-warmed KRB once more. 10–15 islets were then transferred into each of the wells of 12-well plates with 1 ml pre-warmed KRB to start the secretion assays. For all assays, four or more mice of each genotype were used for islet isolation, with islets from two or more mice mixed and examined as 2–3 technical replicas. Total insulin was assayed after ethanol-acid extraction as in (Huang et al., 2018). Insulin was measured with an Elisa kit from ALPCO. Assays from human islets use similar method, except the basal glucose was 3.3 mM. If Pseudo-islets were used, a starting volume of 200 microlitter with 20–30 psuedo-iselts was used due to their small size.

### Targeted DNA methylation and gene expression

Targeted DNA methylation largely followed that in (Liu et al., 2019). Briefly, a plasmid that drives dCas9-DNMT3a-T2A-eGFP expression with CMV promoter/enhancer was purchased from Addgene (#71666). Then a short DNA fragment that drives Syt7 guide RNA (5’-GAACATGTGGGGCTCCAGCG-3’) under the control of a human *U6* promoter was inserted upstream of the CMV enhancer. The resulting plasmid was electroporated into MIN6 cells, which was characterized 5 days after culture. For mRNA assays, cells from three independent plates were FACS-ed based on eGFP expression and studied for mRNA expression using routine reverse transcription coupled with real-time-PCR (see below).

### Real-time RT-PCR-based gene expression assays

Total RNA samples were extracted from isolated islets (from at least three animals) of purified cells (3 batches from independent FACS collection). Pools of cDNA were then prepared using a High Capacity cDNA Reverse Transcription Kit (Thermal Fisher), followed by CYBR-green-based RT-PCR assays (BioRad). The input for each sample was normalized using GAPDH levels. independent plates were sorted based on eGFP expression and studied for mRNA expression using routine reverse transcription coupled with real-time RT-PCR, using GAPDH as a normalizer (oligos used: 5’-AACTTTGGCATTGTGGAAGG-3’ and 5’-GGATGCAGGGATGATGTTCT-3’). Oligos for *Syt7* are (5’-CTTTCGCCTTCGACATACCC-3’ and 5’-GGCTGAGCTTGTCTTTGTCC-3’).

#### In vitro pancreas bud culture assay

As shown in (Liu et al., 2019), E14.5 pancreas buds were dissected and cut into 3–4 pieces. They were then cultured in RPMI1066 supplemented with 10% FBS on filters in an air-liquid interface format, with DMSO or 2 μM AzaC. About 48 hours later, the tissue were prepared for frozen section and used for immunofluorescence assays using antibodies listed in the reagent table. **I**mmunostaining follows standard protocols as in (Liu et al., 2019).

### Maternal diet treatment for bulk RNAseq and labeled cell counting

Six-seven weeks-old female CD1 mice were fed with CD or HFD (Bio-ServTM F3282, with 60% calories from fat compared to 6% in CD) for 2 weeks. They were then crossed with *Ngn3*^*eGFP*^ or MNYI mice under CD or HFD feeding until embryonic collection or birth.

For isolating islet progenitors, pregnant females were dissected at E15.5. Pancreata with eGFP expression were pooled, dissociated, and used for cell isolation via FACS. Embryos from 2–3 litters of mice were pooled and processed as one sample for RNA preparation and sequencing as previously published (Huang et al., 2018). For counting β cells that were labeled with *eYFP* expression in *MNYI* mice, newly born mice and DAM were switched to CD feeding. Pups were then euthanized at P4 for pancreas collection. Islet from those with eYFP and Apple will be used for islet isolation, which were imaged using LSM for cell counting. At least three litters of mice were used for counting.

### Single cell RNA-sequencing and analysis

#### Murine scRNA-seq, single-cell encapsulation and library generation

Dissociated cells from islets were washed and checked for viability with Trypan Blue. Samples with > 95% viability were counted and mixed with 10% K562 cells to evaluate doublet formation rate. Cells were washed using 0.02%BSA in DPBS before resuspension in a solution of DPBS with 15% Optiprep at 100,000 cells/mL before proceeding to inDrops (1CellBio) encapsulation as described in (Klein et al., 2015; Liu et al., 2019; Simmons and Lau, 2022). In brief, cells were flowed into a microfluidic chip where cells were mixed with an RT/Lysis buffer and barcoded capture beads immediately before being portioned into droplets. Cell concentrations and flow rates were carefully controlled to minimize doublets and ensure droplet uniformity. The droplet emulsion was collected into a 1.5 ml tube in fractions of ~ 3,500 estimated captured cells. Droplets then proceeded to UV exposure using a UV Cleaver to release captured oligos from the beads, a 1-hour incubation at 50 °C to facilitate reverse transcription, and finally a 5-minute incubation at 85 °C to stop the reaction. Emulsions were then cooled on ice and demulsified to isolate the aqueous component containing barcoded cellular cDNA, which is stored at −80 °C for library preparation. Libraries were prepared for sequencing utilizing the TruDrop protocol (Southard-Smith et al., 2020). Samples were then sequenced using the Novaseq 6000 (Illumina) in a customized format. For each sample both tdT^+^ and tdT^−^ β cells were processed and sequenced together and contained islets from 3–4 mice. During sequencing we targeted 120 million reads.

### scRNA-seq, alignment, droplet matrix generation, and droplet matrix quality control

Sequences and droplets were aligned and filtered as described in (Osumi-Sutherland et al., 2021). Briefly, the libraries were processed utilizing the DropEst pipeline (Petukhov et al., 2018) (Fig. S2A, B) and the STAR aligner with Ensemble reference genome, CRCh38 release 25 (Dobin et al., 2013). High-quality single-cell-containing droplets were identified employing the dropkick algorithm (Heiser et al., 2021) in conjunction with prior-knowledge in gene expression profiling (Fig. S2B). Normalization, inverse hyperbolic sine transformation, and Z-score scaling was performed on cells as an AnnData object utilizing the Scanpy toolkit (Wolf et al., 2018). These normalized matrices were then projected into two dimensions using a UMAP initialization based on their 50 principal component decomposition. Gene-expression consistency was confirmed and the final selection of cell-containing droplets was made based on quality metrics such as total counts, mitochondrial count percentages, and gene-expression profiles.

### scRNA-seq, count matrix normalization and UMAP visualization

The Scanpy toolkit and numpy functions were used on the raw count data to normalize by median library size, perform log-like transformation with Arcsinh, and standardize each gene using Z-score (Chen et al., 2021; Wolf et al., 2018). Two models of UMAP visualization were employed after normalization: one based on the standard 50 principal component decomposition (as described in *scRNA-seq, alignment, droplet matrix generation, and droplet matrix quality control* section), and another using Harmony-corrected principal components to correct any sample specific batch effects. UMAP visualizations for murine data were generated using the ‘scanpy.tl.umap’ function, while human UMAP visualizations utilized this function or their own embedded x_UMAP profile. Sample numbers were visualized utilizing the same x_UMAP profile to ensure even sample distribution before further analysis (donor_ID Fig. S7). Both human and murine inputs utilized their 50 principal component decompositions. Murine data were integrated with the Harmony algorithm (Korsunsky et al., 2019) which adjusts these principal components.

### scRNA-seq, cell type labeling and unsupervised clustering

Utilizing the scMRMA (Li et al., 2022) algorithm in R, the PanglaoDB was utilized to label major pancreatic cell types. These algorithm-defined labels were then cross-checked with prior-knowledge gene-expression profiles and corrected to label major pancreatic cell types including: α cell, β cells, δ cells, endothelial cells, and enteric neurons. Employing the Scanpy toolkit, the Leiden algorithm single-cell subpopulations were labeled as described in Chen et al (Fig. 2A) (Chen et al., 2021).

### Murine scRNA-seq, pseudobulk analysis

Cells were subset based on the ‘β cell’ identity (as defined by the scRNA-seq, cell type labeling and unsupervised clustering section). Utilizing the Scanpy toolkit, cells were normalized, Arcsinh transformed, then categorized into tdTomato-positive (tdT^+^) and tdTomato-negative (tdT^−^) subsets based on histogram plots of expression with the cutoff for positivity being 0.9. tdT^+^ and tdT^−^ cells were separately averaged by sample before being averaged by sex or in combination then the difference between tdT^+^ and tdT^−^ was determined and numerically sorted, where tdT was the most highly expressed gene across all β cell samples. To identity differentially expressed genes, low-expressing genes were first filtered out (artificially set at Arcsinh-transformed reading below 0.002 per sample, roughly one read in each cell). Paired t-test was then used to identify differentially expressed genes [p < 0.05, with a difference of at least 5% (log-transformed) between the two samples].

### Bioinformatic gene set enrichment analysis

After identification of DEGs, the Database for Annotation, Visualization and Integrated Discovery (DAVID) was primarily utilized for comprehensive functional clustering analysis (Sherman et al., 2022), which generates clusters of gene ontogeny/processes/molecular signature for functional interpretation. In all cases, similar/overlapping pathways or processes were consolidated and summarized for presentation. Only top 15 or those having [−Log10(p value)] > 1.5 (if less than 15 processes) were presented. In addition, we did not include several general terms such as “transcription” and “cancer” in our lists, because their functional implication in β cells is not informative. In addition, we also utilized the pre-ranked DEGs in GO term enrichment and GSEA utilizing the GSEApy package (Fang et al., 2023). Pathway analyses included GO_Biological_Process_2023, WikiPathways_2019_Mouse, KEGG_2019_Mouse, Reactome_2022, and MSIGDB_Hallmark_2020.

### Human scRNA-seq, processing and gene signature scoring

Two publicly available datasets with focuses on adult T2D disease and control β cell populations were selected for analysis (Fig. S7). The data were normalized and transformed as described using leidon and donor ID-based mapping (in the section on *scRNA-seq, cell type labeling and unsupervised clustering*) (Fig. S7A, B, D, E, G, H). We selected a set of 20 functionally known genes upregulated in the murine pseudobulk analysis. These 20 genes were utilized to create a gene signature score employing the ‘scanpy.tl.score_genes’ function as described in (Osumi-Sutherland et al., 2021; Wolf et al., 2018). Each signature was calculated and standardized using a reference sample size of 2000 genes. Scores were subset based on positivity, with ‘population 1’ (Pop. 1) β cells containing a score higher than 0.2 and ‘Pop. 2’ β cells containing a score lower than 0.2 (gene score human Fig. S7C, F, I). To determine if the ‘Pop. 1’ β cell genes were significantly clustered together, rather than randomly, we utilized the package GiniClust3 on the pre-processed data with our selected gene list (Dong and Yuan, 2020). This identified Gini index values and a corresponding p-value, where a p-value < 0.05 indicates the expression of our gene signature is significantly clustered rather than randomly.

### Human scRNA-seq, differential gene-expression and gene set enrichment analysis

Utilizing the Scanpy toolkit, differential testing of gene expression was performed based on population labels (as defined by the Human scRNA-seq, processing and gene signature scoring section). The ‘scanpy.tl.rank_genes_groups’ function was utilized using Wilcoxon rank-sum and only those statistically significant were placed through GSEA. These pre-ranked genes were placed through GSEA using the GSEApy function (as described in the *scRNA-seq, gene set enrichment analysis and differential gene-expression testing* section). Pathways analyzed included GO_Biological_Process_2023, WikiPathways_2019_Human, KEGG_2021_Human, Reactome_2022, and MSigDB_Hallmark_2020.

### scRNA-seq, population quantification and gene-expression analysis

Percent change in human ‘population 1’ β cells was quantified per patient in both control and T2D β cells and plotted using the Matplotlib and Seaborn toolkits. Utilizing the Scipy toolkit, significance was tested using the ‘scipy.stats.ttest_ind’ assuming equal variance. To quantify gene-expression between tdTomato populations, a gene signature score for each gene of interest was calculated utilizing the methods described above (in *Human scRNA-seq, processing and gene signature scoring*). In order to analyze the scores, a scatterplot was utilized to visualize each score and statistical tests were conducted on their distributions. An initial Kruskal-Wallis test was performed and if the null hypothesis was rejected, a post hoc Mann-Whitney U test was conducted. P-values of less than 0.05 were considered significant.

### DNA methylome assays and analysis

**DNA methylome assays largely followed published protocols for** Tagmentation-based whole genome bisulfite sequencing (T-WGBS) (Adey and Shendure, 2012; Barnett et al., 2020; Wang et al., 2013), which relies on a hyperactive Tn5 transposase to fragment DNA while adding adaptor oligo sequences for sequencing (Wang et al., 2013). The genomic DNA prepared from FACS purified β-cell subtypes were used. The key steps for these assays are:

### Transposome Preparation

T-WGBS adapters were prepared by annealing the oligonucleotides in PCR tubes (10μl 100μM Tn5mC-Apt1 oligo, 10μl 100μM Tn5mC1.1- A1block oligo, 80μl nuclease free water). Oligos were incubated in a PCR thermocycler as follows: 95°C for 3 min, 65°C for 3 min, ramp down to 24°C at a rate of − 1°C/second, hold at 24°C. Annealed oligos were then combined with 100 μl glycerol to create a 5μM, 50% glycerol adapter mixture. Transposomes were assembled by combining equal parts purified Tn5 transposase enzyme and annealed oligos, followed by a 25°C incubation for 60 min at room temperature.

### T-WGBS

T-WGBS libraries were prepared as previously reported (Adey and Shendure 2012, Barnentt et al. 2020, Wang et al., 2013). Genomic DNA from purified β-cell subtypes were extracted using a kit from Zymo (D3020). For each stage/sex, at least three batches of cells from FACS (4–7 mice for each batch, at least 30,000 cells) were combined, yielding 100–150 ng DNA. Genomic DNAwas diluted in a 50μl tagmentation reaction (40 ng genomic DNA, 2.5μl transposome assembled with T-WGBS adapters, 10μl 5X Tris-DMF, XuL Nuclease-free (NF) water to obtain a total reaction volume of 50μl) and incubated at 55°C for 8 min in a PCR thermocycler. Tagmentation reactions were then stopped by adding 250μl Zymo DNA binding buffer from the DNA Clean & Concentrator-5 kit (Zymo). Reactions were purified per the manufacturer’s protocol instructions in the DNA Clean & Concentrator-5 kit (Zymo) and eluted in 15μl NF-water. DNA eluate from the tagmentation reaction was used as input into the subsequent oligo replacement and gap repair reaction (11μl DNA eluate, 2μl 10μM Tn5mC-Repl01 oligo, 2μl 10x ampligase buffer, 2μl dNTPs 2.5mM each). This gap repair reaction was assembled in a PCR tube and incubated as follows in a PCR thermocycler: 50°C for 1 minute, 45°C for 10 minutes, ramp down to 37°C at a rate of − 0.1°C/second, hold at 37°C. Upon reaching 37°C, 1μl T4 DNA polymerase and 2.5μl ampligase were both added separately without removing the tube from the thermocycler. The reaction was mixed using a pipette without removal of the tube from thermocycler, minimizing bubbles. The gap repair reaction was subsequently incubated as follows: 37°C for 30 minutes, hold at 4°C. Optionally, 2μl of the gap repair reaction can be reserved for a test PCR amplification to determine library distribution pre-bisulfite conversion. The gap repair reaction was stopped with the addition of 102.5 μl of Zymo DNA binding buffer from the DNA Clean & Concentrator-5 kit (Zymo). Reactions were purified per the manufacturer’s protocol instructions in the DNA Clean & Concentrator-5 kit (Zymo) and eluted in 20.5μl NF-water. Gap repaired, tagmented DNA was subsequently bisulfite converted according to the manufacturer’s protocol instructions using the EZ DNA Methylation-Gold kit (Zymo). For the bisulfite conversion reaction, 20.5μl gap repaired DNA was mixed with 130μl CT conversion reagent in a PCR tube and incubated in a PCR thermocycler as follows: 98°C for 10 min, 64°C for 2.5 hrs, hold at 4°C. Final purification/desulfonation was performed as directed by the kit manufacturer’s protocol instructions. Final elution was in 25μl NF-water. Eluted bisulfite-converted DNA was amplified and barcoded in 50μl PCR reactions (25μl 2x KAPA HiFi HotStart Uracil + ReadyMix, 20μl eluted bisulfite-converted DNA, 1.5μl 10μM i5 index primer, 1.5μl 10μM i7 index primer) as follows: 98°C 45 sec; 10 cycles of 98°C 15 sec, 62°C 30 sec, 72°C 30 sec; final extension 72°C 2 min; hold at 12°C. Post- amplification PCR reactions were purified using the DNA Clean and Concentrator-5 kit (Zymo) and eluted in 22μl NF-water. Library concentrations and size distributions were evaluated using an Agilent 2200 TapeStation with a D5000 screentape. T-WGBS DNA libraries were sequenced at Vanderbilt University’s genomics research core using 2×150bp paired-end reads on the Illumina NovSeq 6000, obtaining approximately 300–500 million reads per library.

### Sequencing Library Processing for T-WGBS Libraries

For each WGBS library, reads were trimmed of their adapters and evaluated for quality control using the TrimGalore Perl script wrapper for Cutadapt and FastQC, respectively (Martin, 2011). Reads were mapped with the MethPipe software’s mapping tool, abismal, to the mm39 genome assembly (Song et al., 2013) (Song et al., 2021). The Preseq software was used to predict library complexities (Daley and Smith, 2013). Methylation analysis of reads, like calling DMRs, was performed using MethPipe (Song et al., 2021). TF motif enrichment analysis of DMRs was performed using the HOMER software. Gene assignments for DMRs were obtained with the web-based tool, GREAT, using default gene annotation protocol with a maximum extension of 1 MB (McLean et al., 2010). In other words, genes that lie right next to each DMR (within up-to 1MB) were identified as potential regulatory targets of the DMRs. Gene ontology analysis was conducted using DAVID as well, with processes/terms/pathways consolidated and presented as for DEGs (Sherman et al., 2022). The BEDtools suite program was used to aid in data manipulation (Quinlan and Hall, 2010).

## Statistical analysis

Data in the figures were represented as mean ± SEM, with individual data point included in most case. Student’s *t*-test was used for statistical comparison between two groups that are normally distributed without repeated measures. For those with repeated measures (e.g., IPGTT), 2way ANOVA was used. To determine if gene enrichment is significant when associating DEGs with DMR-associated genes, Fisher Exact test was used to compare the number of actual associated genes with that predicted from random correlation. In order to compare the over-lap between DMRs and DMR-associated genes at P2 and P60, hypergeometric analysis was used. For all assays, *p*-values of less than 0.05 were considered significant.

## Lead contact

Further information and requests for resources and reagents should be directed to and will be fulfilled by the Lead Contact, Guoqiang.gu@vanderbilt.edu (615–936-3634).

## Material Availability

Plasmids, mouse lines, and original data generated in this study will be available through the lead contact upon reasonable request.

## Data Availability

The raw sequencing and processed data for all samples will be available in the Gene Expression Omnibus (GEO) database. The associated numbers are: GSE254701 for the bulk RNAseq of endocrine progenitor; GSE254955 DNA methylome assays; GSE255135 for the single cell RNAseq data.

## References

[1] Adey A., Shendure J., 2012. Ultra-low-input, tagmentation-based whole-genome bisulfite sequencing. Genome Res 22, 1139–1143. 22466172 10.1101/gr.136242.111 PMC3371708

[2] Aguayo-Mazzucato C., van Haaren M., Mruk M., Lee T.B. Jr., Crawford C., Hollister-Lock J., Sullivan B.A., Johnson J.W., Ebrahimi A., Dreyfuss J.M., Van Deursen J., Weir G.C., Bonner-Weir S., 2017. beta Cell Aging Markers Have Heterogeneous Distribution and Are Induced by Insulin Resistance. Cell Metab 25, 898–910 e895. 28380379 10.1016/j.cmet.2017.03.015 PMC5471618

[3] Avrahami D., Kaestner K.H., 2019. The dynamic methylome of islets in health and disease. Mol Metab 27S, S25–S32. 31500828 10.1016/j.molmet.2019.06.007 PMC6768570

[4] Avrahami D., Li C., Zhang J., Schug J., Avrahami R., Rao S., Stadler M.B., Burger L., Schubeler D., Glaser B., Kaestner K.H., 2015. Aging-Dependent Demethylation of Regulatory Elements Correlates with Chromatin State and Improved beta Cell Function. Cell Metab 22, 619–632. 26321660 10.1016/j.cmet.2015.07.025 PMC4598285

[5] Bader E., Migliorini A., Gegg M., Moruzzi N., Gerdes J., Roscioni S.S., Bakhti M., Brandl E., Irmler M., Beckers J., Aichler M., Feuchtinger A., Leitzinger C., Zischka H., Wang-Sattler R., Jastroch M., Tschop M., Machicao F., Staiger H., Haring H.U., Chmelova H., Chouinard J.A., Oskolkov N., Korsgren O., Speier S., Lickert H., 2016. Identification of proliferative and mature beta-cells in the islets of Langerhans. Nature 535, 430–434. 27398620 10.1038/nature18624

[6] Barnett K.R., Decato B.E., Scott T.J., Hansen T.J., Chen B., Attalla J., Smith A.D., Hodges E., 2020. ATAC-Me Captures Prolonged DNA Methylation of Dynamic Chromatin Accessibility Loci during Cell Fate Transitions. Mol Cell 77, 1350–1364 e1356. 31999955 10.1016/j.molcel.2020.01.004 PMC7169048

[7] Blodgett D.M., Nowosielska A., Afik S., Pechhold S., Cura A.J., Kennedy N.J., Kim S., Kucukural A., Davis R.J., Kent S.C., Greiner D.L., Garber M.G., Harlan D.M., diIorio P., 2015. Novel Observations From Next-Generation RNA Sequencing of Highly Purified Human Adult and Fetal Islet Cell Subsets. Diabetes 64, 3172–3181. 25931473 10.2337/db15-0039 PMC4542439

[8] Bonner-Weir S., Aguayo-Mazzucato C., 2016. Physiology: Pancreatic beta-cell heterogeneity revisited. Nature 535, 365–366. 27398615 10.1038/nature18907

[9] Brasacchio D., Okabe J., Tikellis C., Balcerczyk A., George P., Baker E.K., Calkin A.C., Brownlee M., Cooper M.E., El-Osta A., 2009. Hyperglycemia induces a dynamic cooperativity of histone methylase and demethylase enzymes associated with gene-activating epigenetic marks that coexist on the lysine tail. Diabetes 58, 1229–1236. 19208907 10.2337/db08-1666 PMC2671038

[10] Camunas-Soler J., Dai X.Q., Hang Y., Bautista A., Lyon J., Suzuki K., Kim S.K., Quake S.R., MacDonald P.E., 2020. Patch-Seq Links Single-Cell Transcriptomes to Human Islet Dysfunction in Diabetes. Cell Metab 31, 1017–1031 e1014. 32302527 10.1016/j.cmet.2020.04.005 PMC7398125

[11] Chen B., Scurrah C.R., McKinley E.T., Simmons A.J., Ramirez-Solano M.A., Zhu X., Markham N.O., Heiser C.N., Vega P.N., Rolong A., Kim H., Sheng Q., Drewes J.L., Zhou Y., Southard-Smith A.N., Xu Y., Ro J., Jones A.L., Revetta F., Berry L.D., Niitsu H., Islam M., Pelka K., Hofree M., Chen J.H., Sarkizova S., Ng K., Giannakis M., Boland G.M., Aguirre A.J., Anderson A.C., Rozenblatt-Rosen O., Regev A., Hacohen N., Kawasaki K., Sato T., Goettel J.A., Grady W.M., Zheng W., Washington M.K., Cai Q., Sears C.L., Goldenring J.R., Franklin J.L., Su T., Huh W.J., Vandekar S., Roland J.T., Liu Q., Coffey R.J., Shrubsole M.J., Lau K.S., 2021. Differential pre-malignant programs and microenvironment chart distinct paths to malignancy in human colorectal polyps. Cell 184, 6262–6280 e6226. 34910928 10.1016/j.cell.2021.11.031 PMC8941949

[12] Chiou J., Zeng C., Cheng Z., Han J.Y., Schlichting M., Miller M., Mendez R., Huang S., Wang J., Sui Y., Deogaygay A., Okino M.L., Qiu Y., Sun Y., Kudtarkar P., Fang R., Preissl S., Sander M., Gorkin D.U., Gaulton K.J., 2021. Single-cell chromatin accessibility identifies pancreatic islet cell type- and state-specific regulatory programs of diabetes risk. Nat Genet 53, 455–466. 33795864 10.1038/s41588-021-00823-0 PMC9037575

[13] Daley T., Smith A.D., 2013. Predicting the molecular complexity of sequencing libraries. Nat Methods 10, 325–327. 23435259 10.1038/nmeth.2375 PMC3612374

[14] Dhar G.A., Saha S., Mitra P., Nag Chaudhuri R., 2021. DNA methylation and regulation of gene expression: Guardian of our health. Nucleus (Calcutta) 64, 259–270. 34421129 10.1007/s13237-021-00367-y PMC8366481

[15] Dhawan S., Georgia S., Tschen S.I., Fan G., Bhushan A., 2011. Pancreatic beta cell identity is maintained by DNA methylation-mediated repression of Arx. Dev Cell 20, 419–429. 21497756 10.1016/j.devcel.2011.03.012 PMC3086024

[16] Dhawan S., Tschen S.I., Zeng C., Guo T., Hebrok M., Matveyenko A., Bhushan A., 2015. DNA methylation directs functional maturation of pancreatic beta cells. J Clin Invest 125, 2851–2860. 26098213 10.1172/JCI79956 PMC4563682

[17] Dickerson M.T., Dadi P.K., Altman M.K., Verlage K.R., Thorson A.S., Jordan K.L., Vierra N.C., Amarnath G., Jacobson D.A., 2019. Glucose-mediated inhibition of calcium-activated potassium channels limits alpha-cell calcium influx and glucagon secretion. Am J Physiol Endocrinol Metab 316, E646–E659. 30694690 10.1152/ajpendo.00342.2018 PMC6482666

[18] Dobin A., Davis C.A., Schlesinger F., Drenkow J., Zaleski C., Jha S., Batut P., Chaisson M., Gingeras T.R., 2013. STAR: ultrafast universal RNA-seq aligner. Bioinformatics 29, 15–21. 23104886 10.1093/bioinformatics/bts635 PMC3530905

[19] Doherty J.E., Huye L.E., Yusa K., Zhou L., Craig N.L., Wilson M.H., 2012. Hyperactive piggyBac gene transfer in human cells and in vivo. Hum Gene Ther 23, 311–320. 21992617 10.1089/hum.2011.138 PMC3300075

[20] Dolai S., Xie L., Zhu D., Liang T., Qin T., Xie H., Kang Y., Chapman E.R., Gaisano H.Y., 2016. Synaptotagmin-7 Functions to Replenish Insulin Granules for Exocytosis in Human Islet beta-Cells. Diabetes 65, 1962–1976. 27207520 10.2337/db15-1436 PMC5384637

[21] Dominguez-Gutierrez G., Xin Y., Gromada J., 2019. Heterogeneity of human pancreatic beta-cells. Mol Metab 27S, S7–S14. 31500834 10.1016/j.molmet.2019.06.015 PMC6768494

[22] Dong R., Yuan G.C., 2020. GiniClust3: a fast and memory-efficient tool for rare cell type identification. BMC Bioinformatics 21, 158. 32334526 10.1186/s12859-020-3482-1 PMC7183612

[23] Dorrell C., Schug J., Canaday P.S., Russ H.A., Tarlow B.D., Grompe M.T., Horton T., Hebrok M., Streeter P.R., Kaestner K.H., Grompe M., 2016. Human islets contain four distinct subtypes of beta cells. Nat Commun 7, 11756. 27399229 10.1038/ncomms11756 PMC4942571

[24] Dror E., Fagnocchi L., Wegert V., Apostle S., Grimaldi B., Gruber T., Panzeri I., Heyne S., Hoffler K.D., Kreiner V., Ching R., Tsai-Hsiu Lu T., Semwal A., Johnson B., Senapati P., Lempradl A., Schones D., Imhof A., Shen H., Pospisilik J.A., 2023. Epigenetic dosage identifies two major and functionally distinct beta cell subtypes. Cell Metab 35, 821–836 e827. 36948185 10.1016/j.cmet.2023.03.008 PMC10160009

[25] Elsakr J.M., Dunn J.C., Tennant K., Zhao S.K., Kroeten K., Pasek R.C., Takahashi D.L., Dean T.A., Velez Edwards D.R., McCurdy C.E., Aagaard K.M., Powers A.C., Friedman J.E., Kievit P., Gannon M., 2019. Maternal Western-style diet affects offspring islet composition and function in a non-human primate model of maternal over-nutrition. Mol Metab 25, 73–82. 31036449 10.1016/j.molmet.2019.03.010 PMC6599455

[26] Ernst P., Kim S., Yang Z., Liu X.M., Zhou L., 2023. Characterization of the far-red fluorescent probe MitoView 633 for dynamic mitochondrial membrane potential measurement. Front Physiol 14, 1257739. 37936577 10.3389/fphys.2023.1257739 PMC10627182

[27] Fang Z., Liu X., Peltz G., 2023. GSEApy: a comprehensive package for performing gene set enrichment analysis in Python. Bioinformatics 39. 10.1093/bioinformatics/btac757 PMC9805564 36426870

[28] Fernandez-de-Cossio-Diaz J., Mulet R., Vazquez A., 2019. Cell population heterogeneity driven by stochastic partition and growth optimality. Sci Rep 9, 9406. 31253860 10.1038/s41598-019-45882-w PMC6599024

[29] Finegood D.T., Scaglia L., Bonner-Weir S., 1995. Dynamics of beta-cell mass in the growing rat pancreas. Estimation with a simple mathematical model. Diabetes 44, 249–256. 7883109 10.2337/diab.44.3.249

[30] Friedlander M.S.H., Nguyen V.M., Kim S.K., Bevacqua R.J., 2021. Pancreatic Pseudoislets: An Organoid Archetype for Metabolism Research. Diabetes 70, 1051–1060. 33947722 10.2337/db20-1115 PMC8343609

[31] Friedman J.E., 2018. Developmental Programming of Obesity and Diabetes in Mouse, Monkey, and Man in 2018: Where Are We Headed? Diabetes 67, 2137–2151. 30348820 10.2337/dbi17-0011 PMC6198344

[32] Gannon M., Kulkarni R.N., Tse H.M., Mauvais-Jarvis F., 2018. Sex differences underlying pancreatic islet biology and its dysfunction. Mol Metab 15, 82–91. 29891438 10.1016/j.molmet.2018.05.017 PMC6066785

[33] Gauthier B.R., Wollheim C.B., 2008. Synaptotagmins bind calcium to release insulin. Am J Physiol Endocrinol Metab 295, E1279–1286. 18713958 10.1152/ajpendo.90568.2008

[34] Golson M.L., Dunn J.C., Maulis M.F., Dadi P.K., Osipovich A.B., Magnuson M.A., Jacobson D.A., Gannon M., 2015. Activation of FoxM1 Revitalizes the Replicative Potential of Aged beta-Cells in Male Mice and Enhances Insulin Secretion. Diabetes 64, 3829–3838. 26251404 10.2337/db15-0465 PMC4613976

[35] Gu C., Stein G.H., Pan N., Goebbels S., Hornberg H., Nave K.A., Herrera P., White P., Kaestner K.H., Sussel L., Lee J.E., 2010. Pancreatic beta cells require NeuroD to achieve and maintain functional maturity. Cell Metab 11, 298–310. 20374962 10.1016/j.cmet.2010.03.006 PMC2855640

[36] Gu G., Dubauskaite J., Melton D.A., 2002. Direct evidence for the pancreatic lineage: NGN3+ cells are islet progenitors and are distinct from duct progenitors. Development 129, 2447–2457. 11973276 10.1242/dev.129.10.2447

[37] Gupta R.K., Gao N., Gorski R.K., White P., Hardy O.T., Rafiq K., Brestelli J.E., Chen G., Stoeckert C.J. Jr., Kaestner K.H., 2007. Expansion of adult beta-cell mass in response to increased metabolic demand is dependent on HNF-4alpha. Genes Dev 21, 756–769. 17403778 10.1101/gad.1535507 PMC1838528

[38] Hall E., Dekker Nitert M., Volkov P., Malmgren S., Mulder H., Bacos K., Ling C., 2018. The effects of high glucose exposure on global gene expression and DNA methylation in human pancreatic islets. Mol Cell Endocrinol 472, 57–67. 29183809 10.1016/j.mce.2017.11.019

[39] Hall E., Jonsson J., Ofori J.K., Volkov P., Perfilyev A., Dekker Nitert M., Eliasson L., Ling C., Bacos K., 2019. Glucolipotoxicity Alters Insulin Secretion via Epigenetic Changes in Human Islets. Diabetes 68, 1965–1974. 31420409 10.2337/db18-0900

[40] Heiser C.N., Wang V.M., Chen B., Hughey J.J., Lau K.S., 2021. Automated quality control and cell identification of droplet-based single-cell data using dropkick. Genome Res 31, 1742–1752. 33837131 10.1101/gr.271908.120 PMC8494217

[41] Hu R., Walker E., Huang C., Xu Y., Weng C., Erickson G.E., Coldren A., Yang X., Brissova M., Kaverina I., Balamurugan A.N., Wright C.V.E., Li Y., Stein R., Gu G., 2020. Myt Transcription Factors Prevent Stress-Response Gene Overactivation to Enable Postnatal Pancreatic beta Cell Proliferation, Function, and Survival. Dev Cell 53, 390–405 e310. 32359405 10.1016/j.devcel.2020.04.003 PMC7278035

[42] Huang C., Walker E.M., Dadi P.K., Hu R., Xu Y., Zhang W., Sanavia T., Mun J., Liu J., Nair G.G., Tan H.Y.A., Wang S., Magnuson M.A., Stoeckert C.J. Jr., Hebrok M., Gannon M., Han W., Stein R., Jacobson D.A., Gu G., 2018. Synaptotagmin 4 Regulates Pancreatic beta Cell Maturation by Modulating the Ca(2+) Sensitivity of Insulin Secretion Vesicles. Dev Cell 45, 347–361 e345. 29656931 10.1016/j.devcel.2018.03.013 PMC5962294

[43] Iezzi M., Kouri G., Fukuda M., Wollheim C.B., 2004. Synaptotagmin V and IX isoforms control Ca2+ - dependent insulin exocytosis. J Cell Sci 117, 3119–3127. 15190121 10.1242/jcs.01179

[44] Jensen T.I., Mikkelsen N.S., Gao Z., Fosselteder J., Pabst G., Axelgaard E., Laustsen A., Konig S., Reinisch A., Bak R.O., 2021. Targeted regulation of transcription in primary cells using CRISPRa and CRISPRi. Genome Res 31, 2120–2130. 34407984 10.1101/gr.275607.121 PMC8559706

[45] Johnston N.R., Mitchell R.K., Haythorne E., Pessoa M.P., Semplici F., Ferrer J., Piemonti L., Marchetti P., Bugliani M., Bosco D., Berishvili E., Duncanson P., Watkinson M., Broichhagen J., Trauner D., Rutter G.A., Hodson D.J., 2016. Beta Cell Hubs Dictate Pancreatic Islet Responses to Glucose. Cell Metab 24, 389–401. 27452146 10.1016/j.cmet.2016.06.020 PMC5031557

[46] Kaestner K.H., Powers A.C., Naji A., Consortium H., Atkinson M.A., 2019. NIH Initiative to Improve Understanding of the Pancreas, Islet, and Autoimmunity in Type 1 Diabetes: The Human Pancreas Analysis Program (HPAP). Diabetes 68, 1394–1402. 31127054 10.2337/db19-0058 PMC6609987

[47] Kalwat M.A., Thurmond D.C., 2013. Signaling mechanisms of glucose-induced F-actin remodeling in pancreatic islet beta cells. Exp Mol Med 45, e37. 23969997 10.1038/emm.2013.73 PMC3789261

[48] Katsumoto K., Yennek S., Chen C., Silva L.F.D., Traikov S., Sever D., Azad A., Shan J., Vainio S., Ninov N., Speier S., Grapin-Botton A., 2022. Wnt4 is heterogeneously activated in maturing beta-cells to control calcium signaling, metabolism and function. Nat Commun 13, 6255. 36271049 10.1038/s41467-022-33841-5 PMC9587236

[49] Klein A.M., Mazutis L., Akartuna I., Tallapragada N., Veres A., Li V., Peshkin L., Weitz D.A., Kirschner M.W., 2015. Droplet barcoding for single-cell transcriptomics applied to embryonic stem cells. Cell 161, 1187–1201. 26000487 10.1016/j.cell.2015.04.044 PMC4441768

[50] Korsunsky I., Millard N., Fan J., Slowikowski K., Zhang F., Wei K., Baglaenko Y., Brenner M., Loh P.R., Raychaudhuri S., 2019. Fast, sensitive and accurate integration of single-cell data with Harmony. Nat Methods 16, 1289–1296. 31740819 10.1038/s41592-019-0619-0 PMC6884693

[51] Laakso M., Fernandes Silva L., 2022. Genetics of Type 2 Diabetes: Past, Present, and Future. Nutrients 14. 10.3390/nu14153201 PMC9370092 35956377

[52] Lee S., Zhang J., Saravanakumar S., Flisher M.F., Grimm D.R., van der Meulen T., Huising M.O., 2021. Virgin beta-Cells at the Neogenic Niche Proliferate Normally and Mature Slowly. Diabetes 70, 1070–1083. 33563657 10.2337/db20-0679 PMC8173805

[53] Li J., Sheng Q., Shyr Y., Liu Q., 2022. scMRMA: single cell multiresolution marker-based annotation. Nucleic Acids Res 50, e7. 34648021 10.1093/nar/gkab931 PMC8789072

[54] Li Y., Saldanha S.N., Tollefsbol T.O., 2014. Impact of epigenetic dietary compounds on transgenerational prevention of human diseases. AAPS J 16, 27–36. 24114450 10.1208/s12248-013-9538-7 PMC3877417

[55] Liu J., Banerjee A., Herring C.A., Attalla J., Hu R., Xu Y., Shao Q., Simmons A.J., Dadi P.K., Wang S., Jacobson D.A., Liu B., Hodges E., Lau K.S., Gu G., 2019. Neurog3-Independent Methylation Is the Earliest Detectable Mark Distinguishing Pancreatic Progenitor Identity. Dev Cell 48, 49–63 e47. 30620902 10.1016/j.devcel.2018.11.048 PMC6327977

[56] Lorberbaum D.S., Sussel L., 2017. Gotta Have GATA for Human Pancreas Development. Cell Stem Cell 20, 577–579. 28475878 10.1016/j.stem.2017.04.004

[57] Martin M., 2011. Cutadapt Removes Adapter Sequences From High-Throughput Sequencing Reads. EMBnet.journal 17.

[58] Mawla A.M., Huising M.O., 2019. Navigating the Depths and Avoiding the Shallows of Pancreatic Islet Cell Transcriptomes. Diabetes 68, 1380–1393. 31221802 10.2337/dbi18-0019 PMC6609986

[59] McLean C.Y., Bristor D., Hiller M., Clarke S.L., Schaar B.T., Lowe C.B., Wenger A.M., Bejerano G., 2010. GREAT improves functional interpretation of cis-regulatory regions. Nat Biotechnol 28, 495–501. 20436461 10.1038/nbt.1630 PMC4840234

[60] Mosser R.E., Maulis M.F., Moulle V.S., Dunn J.C., Carboneau B.A., Arasi K., Pappan K., Poitout V., Gannon M., 2015. High-fat diet-induced beta-cell proliferation occurs prior to insulin resistance in C57Bl/6J male mice. Am J Physiol Endocrinol Metab 308, E573–582. 25628421 10.1152/ajpendo.00460.2014 PMC4385873

[61] O’Hagan S., Wright Muelas M., Day P.J., Lundberg E., Kell D.B., 2018. GeneGini: Assessment via the Gini Coefficient of Reference “Housekeeping” Genes and Diverse Human Transporter Expression Profiles. Cell Syst 6, 230–244 e231. 29428416 10.1016/j.cels.2018.01.003 PMC5840522

[62] Ofori J.K., Karagiannopoulos A., Barghouth M., Nagao M., Andersson M.E., Salunkhe V.A., Zhang E., Wendt A., Eliasson L., 2022. The highly expressed calcium-insensitive synaptotagmin-11 and synaptotagmin-13 modulate insulin secretion. Acta Physiol (Oxf) 236, e13857. 35753051 10.1111/apha.13857 PMC9541707

[63] Osumi-Sutherland D., Xu C., Keays M., Levine A.P., Kharchenko P.V., Regev A., Lein E., Teichmann S.A., 2021. Cell type ontologies of the Human Cell Atlas. Nat Cell Biol 23, 1129–1135. 34750578 10.1038/s41556-021-00787-7

[64] Papizan J.B., Singer R.A., Tschen S.I., Dhawan S., Friel J.M., Hipkens S.B., Magnuson M.A., Bhushan A., Sussel L., 2011. Nkx2.2 repressor complex regulates islet beta-cell specification and prevents beta-to-alpha-cell reprogramming. Genes Dev 25, 2291–2305. 22056672 10.1101/gad.173039.111 PMC3219233

[65] Parveen N., Wang J.K., Bhattacharya S., Cuala J., Rajkumar M.S., Butler A.E., Wu X., Shih H.P., Georgia S.K., Dhawan S., 2023. DNA Methylation-Dependent Restriction of Tyrosine Hydroxylase Contributes to Pancreatic beta-Cell Heterogeneity. Diabetes 72, 575–589. 36607262 10.2337/db22-0506 PMC10130487

[66] Petukhov V., Guo J., Baryawno N., Severe N., Scadden D.T., Samsonova M.G., Kharchenko P.V., 2018. dropEst: pipeline for accurate estimation of molecular counts in droplet-based single-cell RNA-seq experiments. Genome Biol 19, 78. 29921301 10.1186/s13059-018-1449-6 PMC6010209

[67] Pipeleers D., De Mesmaeker I., Robert T., Van Hulle F., 2017. Heterogeneity in the Beta-Cell Population: a Guided Search Into Its Significance in Pancreas and in Implants. Curr Diab Rep 17, 86. 28812213 10.1007/s11892-017-0925-9 PMC5557868

[68] Piper K., Brickwood S., Turnpenny L.W., Cameron I.T., Ball S.G., Wilson D.I., Hanley N.A., 2004. Beta cell differentiation during early human pancreas development. J Endocrinol 181, 11–23. 15072563 10.1677/joe.0.1810011

[69] Quinlan A.R., Hall I.M., 2010. BEDTools: a flexible suite of utilities for comparing genomic features. Bioinformatics 26, 841–842. 20110278 10.1093/bioinformatics/btq033 PMC2832824

[70] Ramond C., Glaser N., Berthault C., Ameri J., Kirkegaard J.S., Hansson M., Honore C., Semb H., Scharfmann R., 2017. Reconstructing human pancreatic differentiation by mapping specific cell populations during development. Elife 6. 10.7554/eLife.27564 PMC5540466 28731406

[71] Reizel Y., Morgan A., Gao L., Schug J., Mukherjee S., Garcia M.F., Donahue G., Baur J.A., Zaret K.S., Kaestner K.H., 2021. FoxA-dependent demethylation of DNA initiates epigenetic memory of cellular identity. Dev Cell 56, 602–612 e604. 33636105 10.1016/j.devcel.2021.02.005 PMC8129911

[72] Rubio-Navarro A., Gomez-Banoy N., Stoll L., Dundar F., Mawla A.M., Ma L., Cortada E., Zumbo P., Li A., Reiterer M., Montoya-Oviedo N., Homan E.A., Imai N., Gilani A., Liu C., Naji A., Yang B., Chong A.C.N., Cohen D.E., Chen S., Cao J., Pitt G.S., Huising M.O., Betel D., Lo J.C., 2023. A beta cell subset with enhanced insulin secretion and glucose metabolism is reduced in type 2 diabetes. Nat Cell Biol 25, 565–578. 36928765 10.1038/s41556-023-01103-1 PMC10449536

[73] Salomon D., Meda P., 1986. Heterogeneity and contact-dependent regulation of hormone secretion by individual B cells. Exp Cell Res 162, 507–520. 3510882 10.1016/0014-4827(86)90354-x

[74] Scott T.J., Hansen T.J., McArthur E., Hodges E., 2023. Cross-tissue patterns of DNA hypomethylation reveal genetically distinct histories of cell development. BMC Genomics 24, 623. 37858046 10.1186/s12864-023-09622-9 PMC10588161

[75] Sherman B.T., Hao M., Qiu J., Jiao X., Baseler M.W., Lane H.C., Imamichi T., Chang W., 2022. DAVID: a web server for functional enrichment analysis and functional annotation of gene lists (2021 update). Nucleic Acids Res 50, W216–W221. 35325185 10.1093/nar/gkac194 PMC9252805

[76] Simmons A.J., Lau K.S., 2022. Dissociation and inDrops microfluidic encapsulation of human gut tissues for single-cell atlasing studies. STAR Protoc 3, 101570. 35880121 10.1016/j.xpro.2022.101570 PMC9307676

[77] Song Q., Decato B., Hong E.E., Zhou M., Fang F., Qu J., Garvin T., Kessler M., Zhou J., Smith A.D., 2013. A reference methylome database and analysis pipeline to facilitate integrative and comparative epigenomics. PLoS One 8, e81148. 24324667 10.1371/journal.pone.0081148 PMC3855694

[78] Southard-Smith A.N., Simmons A.J., Chen B., Jones A.L., Ramirez Solano M.A., Vega P.N., Scurrah C.R., Zhao Y., Brenan M.J., Xuan J., Shrubsole M.J., Porter E.B., Chen X., Brenan C.J.H., Liu Q., Quigley L.N.M., Lau K.S., 2020. Dual indexed library design enables compatibility of in-Drop single-cell RNA-sequencing with exAMP chemistry sequencing platforms. BMC Genomics 21, 456. 32616006 10.1186/s12864-020-06843-0 PMC7331155

[79] Stancill J.S., Osipovich A.B., Cartailler J.P., Magnuson M.A., 2019. Transgene-associated human growth hormone expression in pancreatic beta-cells impairs identification of sex-based gene expression differences. Am J Physiol Endocrinol Metab 316, E196–E209. 30532991 10.1152/ajpendo.00229.2018 PMC6397359

[80] Talchai C., Xuan S., Lin H.V., Sussel L., Accili D., 2012. Pancreatic beta cell dedifferentiation as a mechanism of diabetic beta cell failure. Cell 150, 1223–1234. 22980982 10.1016/j.cell.2012.07.029 PMC3445031

[81] Thompson B., Satin L.S., 2021. Beta-Cell Ion Channels and Their Role in Regulating Insulin Secretion. Compr Physiol 11, 1–21. 34636409 10.1002/cphy.c210004 PMC8935893

[82] Thompson P.J., Shah A., Ntranos V., Van Gool F., Atkinson M., Bhushan A., 2019. Targeted Elimination of Senescent Beta Cells Prevents Type 1 Diabetes. Cell Metab 29, 1045–1060 e1010. 30799288 10.1016/j.cmet.2019.01.021

[83] van der Meulen T., Mawla A.M., DiGruccio M.R., Adams M.W., Nies V., Dolleman S., Liu S., Ackermann A.M., Caceres E., Hunter A.E., Kaestner K.H., Donaldson C.J., Huising M.O., 2017. Virgin Beta Cells Persist throughout Life at a Neogenic Niche within Pancreatic Islets. Cell Metab 25, 911–926 e916. 28380380 10.1016/j.cmet.2017.03.017 PMC8586897

[84] Wadhwa P.D., Buss C., Entringer S., Swanson J.M., 2009. Developmental origins of health and disease: brief history of the approach and current focus on epigenetic mechanisms. Semin Reprod Med 27, 358–368. 19711246 10.1055/s-0029-1237424 PMC2862635

[85] Wang Q., Gu L., Adey A., Radlwimmer B., Wang W., Hovestadt V., Bahr M., Wolf S., Shendure J., Eils R., Plass C., Weichenhan D., 2013. Tagmentation-based whole-genome bisulfite sequencing. Nat Protoc 8, 2022–2032. 24071908 10.1038/nprot.2013.118

[86] Weng C., Gu A., Zhang S., Lu L., Ke L., Gao P., Liu X., Wang Y., Hu P., Plummer D., MacDonald E., Zhang S., Xi J., Lai S., Leskov K., Yuan K., Jin F., Li Y., 2023. Single cell multiomic analysis reveals diabetes-associated beta-cell heterogeneity driven by HNF1A. Nat Commun 14, 5400. 37669939 10.1038/s41467-023-41228-3 PMC10480445

[87] Wolf F.A., Angerer P., Theis F.J., 2018. SCANPY: large-scale single-cell gene expression data analysis. Genome Biol 19, 15. 29409532 10.1186/s13059-017-1382-0 PMC5802054

[88] Wortham M., Liu F., Harrington A.R., Fleischman J.Y., Wallace M., Mulas F., Mallick M., Vinckier N.K., Cross B.R., Chiou J., Patel N.A., Sui Y., McGrail C., Jun Y., Wang G., Jhala U.S., Schule R., Shirihai O.S., Huising M.O., Gaulton K.J., Metallo C.M., Sander M., 2023. Nutrient regulation of the islet epigenome controls adaptive insulin secretion. J Clin Invest 133. 10.1172/JCI165208 PMC10104905 36821378

[89] Wu B., Wei S., Petersen N., Ali Y., Wang X., Bacaj T., Rorsman P., Hong W., Sudhof T.C., Han W., 2015. Synaptotagmin-7 phosphorylation mediates GLP-1-dependent potentiation of insulin secretion from beta-cells. Proceedings of the National Academy of Sciences of the United States of America 112, 9996–10001. 26216970 10.1073/pnas.1513004112 PMC4538675

[90] Xin Y., Dominguez Gutierrez G., Okamoto H., Kim J., Lee A.H., Adler C., Ni M., Yancopoulos G.D., Murphy A.J., Gromada J., 2018. Pseudotime Ordering of Single Human beta-Cells Reveals States of Insulin Production and Unfolded Protein Response. Diabetes 67, 1783–1794. 29950394 10.2337/db18-0365

[91] Zanetti M., Barazzoni R., Kiwanuka E., Vettore M., Vedovato M., Tessari P., 2020. Accelerated whole-body protein catabolism in subjects with type 2 Diabetes Mellitus and albuminuria. PLoS One 15, e0243638. 33332405 10.1371/journal.pone.0243638 PMC7746191

[92] Zeng C., Mulas F., Sui Y., Guan T., Miller N., Tan Y., Liu F., Jin W., Carrano A.C., Huising M.O., Shirihai O.S., Yeo G.W., Sander M., 2017. Pseudotemporal Ordering of Single Cells Reveals Metabolic Control of Postnatal beta Cell Proliferation. Cell Metab 25, 1160–1175 e1111. 28467932 10.1016/j.cmet.2017.04.014 PMC5501713

[93] Zhang Z., Gao Y., Meng Z.X., 2022. Transcriptional control of pancreatic beta-cell identity and plasticity during the pathogenesis of type 2 diabetes. J Genet Genomics 49, 316–328. 35292418 10.1016/j.jgg.2022.03.002

